# Solving the OH + Glyoxal Problem: A Complete Theoretical Description of Post-Transition-State Energy Deposition in Activated Systems

**DOI:** 10.1021/acs.jpca.3c07823

**Published:** 2024-02-20

**Authors:** Robin Shannon, Mark A. Blitz, Paul W. Seakins

**Affiliations:** † School of Chemistry, University of Leeds, Leeds LS2 9JT, U.K.; ‡ National Centre for Atmospheric Science, University of Leeds, Leeds LS2 9JT, U.K.

## Abstract

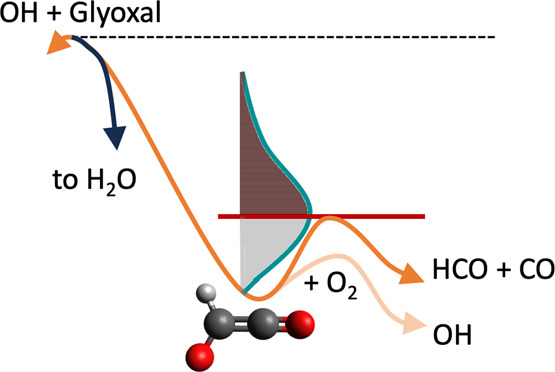

Activated chemistry in coupled reaction systems has broadened our understanding of the chemical kinetics. In the case of intermediates formed in gas phase abstraction reactions (e.g., OH + HC(O)C(O)H (glyoxal) →HC(O)CO + H_2_O), it is particularly crucial to understand how the reaction energy is partitioned between product species as this determines the propensity for a given product to undergo “prompt” dissociation (e.g., HC(O)CO → HCO + CO) before the excess reaction energy is removed. An example of such an activated system is the OH + glyoxal + O_2_ coupled reaction system. In this work, we develop a molecular dynamics pipeline, which, combined with a master equation analysis, accurately models previous experimental measurements. This new work resolves previous complexities and discrepancies from earlier master equation modeling for this reaction system. The detailed molecular dynamics approach employed here is a powerful new tool for modeling challenging activated reaction systems.

## 1 Introduction

Gas phase reaction kinetics underpins the chemistry of a multitude of key environments, from planetary atmospheres^1^ to combustion engines.^2^ Experimental and theoretical techniques allow us to study the kinetics and mechanism of gas phase reactions in unprecedented detail, but they have also illuminated a great deal of complexity regarding the importance of chemical activation when simple reactions are coupled together in real environments. A key paper in *Science* demonstrated that in the atmospheric reaction of OH and acetylene, the energy deposited in the HOC_2_H_2_ chemically activated addition product persists long enough for molecular oxygen to undergo reactive collisions with these “hot” species as they cool.^3^ Nonthermal effects arising from bimolecular reaction of energetically excited products or intermediates have been shown to be crucial in a range of systems.^4−11^ This body of work demonstrates that even in simple, well-studied, prototypical reactions such as that between OH + acetylene, a richness of chemistry can open up when the assumption of rapidly thermalized products is discounted. Increasingly routinely, it is necessary to couple reactions together in a master equation to examine multiple sequential processes in microscopic detail.^7−9,11,12^

Abstraction reactions^5,10,13,14^ present even more complexity than the addition reactions considered in the OH/C_2_H_2_/O_2_ system discussed above. For addition reactions, the entire exothermicity of the reaction is deposited into the addition product; however, for abstraction reactions such as the OH + glyoxal reaction (R1) discussed in this paper:
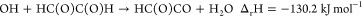
R1the partitioning of the reaction exothermicity between the two products needs to be considered. If sufficient energy is deposited into the HC(O)CO fragment, then chemically activated decomposition (R2) can occur before the interception of the HC(O)CO radical by other species, for example, the reaction with O_2_ in the atmospheric oxidation of glyoxal (R3/R4).
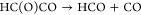
R2
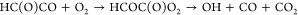
R3/R4Isomerization of HCOC(O)O_2_ leads to COC(O)OOH and then to OH. Observation of the yield of OH is experimentally how the degree of HCOCO fragmentation is monitored.^5,14^ In the atmosphere, R2 forms the HCO radical, which rapidly reacts with molecular oxygen (R5)
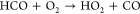
R5

The combination R2/R5 forms the relatively unreactive HO_2_ radical and 2CO, whereas reactions R3/R4 lead to reactive OH radicals and CO + CO_2_.

The importance of nonequilibrium chemistry and relaxation dynamics postreaction is not confined to the gas phase. Several experimental and dynamical studies have observed nonthermal phenomena in solution phase reactions,^15−17^ and one study has demonstrated competition between chemical reaction and energy relaxation in surface chemistry relevant to diamond etching.^18^ These studies show that nonthermal chemistry can prevail even into phases with extremely efficient energy transfer.

Despite the established importance of nonequilibrium chemistry, theoretical studies are challenging to perform. Going back as far as Polanyi,^19^ concepts such as “early” and “late” barriers imply that partitioning of energy postreaction is a dynamical rather than a statistical phenomenon and indeed, our previous work^5^ has shown a statistical “prior” distribution model to poorly describe the kinetics of the OH + glyoxal (+O_2_) reaction. In this previous work, it was necessary to substantially modify the “prior” distribution by a correction factor obtained through fitting to experimental data. This correction was purely empirical, and consequently, our statistical model for the system could not offer any insights into the system from an *a priori* perspective. Furthermore, the modified “prior” distribution did not reproduce an apparent temperature dependence of the energy partitioning that was observed experimentally.

This previous work highlights a need for predictive calculations of energy partitioning postreaction; however, the dynamic nature of these processes requires trajectory calculations, and these are expensive to perform routinely given the relatively large amount of accurate potential energy information required when compared to a statistical model. In fact, given the predictive quality of recent rate theory calculations, a quantitative description of postreaction energy distribution between two fragments is one of the largest remaining areas of uncertainty when performing master equation simulations of complex reactions.^20^

Despite the importance of postreaction energy partitioning, only a relatively small number of dynamics studies have been performed in this field. Importantly, recent work from Labbe et al. and Goldsmith and co-workers^6,21,22^ has started incorporating molecular dynamics (MD) calculations into coupled reaction problems in combustion systems, and work from Danilack and Goldsmith^22^ has proposed convenient statistical expressions which approximate full dynamical studies. However, these studies have to date been performed on a relatively small number of systems and all used quasi-classical microcanonical sampling (QCMS) schemes^23,24^ at the reaction dividing surface. It should be noted that vibrational dynamics is a thriving area of research in its own right^25,26^ but in this work we are particularly focused upon postreaction dynamics for larger systems in the context of activated kinetics and it is this close coupling between MD and statistical rate theory which has to date received little attention.

In this work, we utilize a combination of theoretical techniques to produce an *apriori* model for the complex nonthermal chemistry in the OH + glyoxal reaction. This system is a particularly sensitive benchmark for the trajectory approaches that are emerging. This paper is organized as follows: In Section 2, we set out a generalizable theoretical workflow for treating postreaction vibrational dynamics and deal with each of the theoretical techniques in turn. Then in Section 3, we present results for the OH + glyoxal system. The results section is divided into two parts. Section 3.1 concerns the distribution of reaction energy among the products from MD simulations, while Section 3.2 incorporates the MD distributions into a full master equation model of the OH + glyoxal system to compare our theoretical model with experiment. Finally, Section 4 presents some conclusions.

## 2 Methodology

Key to this work is the development of a convenient theoretical workflow for describing postreaction vibrational dynamics and the distribution of reaction energy among product fragments. The workflow used to treat the postreaction dynamics involves three connected steps. These are summarized below and then will be individually addressed in more detail. The main considerations are:

**Generating an efficient (neural net) force field for the system**: A large amount of *ab initio* data were used to fit a machine learning potential using the PhysNet code of Unke and Meuwly^27,28^ to allow for accurate and efficient “on-the-fly” force calls during the MD runs.

**Equilibrated transition state sampling (ETSS)/accelerating bimolecular reaction**: In this work, the majority of the dynamics simulations are initiated at the reaction saddle point using our new “equilibrated transition state sampling” (ETSS) method, where initial conditions are generated by fixing the breaking and forming bonds in the transition state and allowing all other degrees of freedom to equilibrate in the usual classical manner. We were also able to perform some dynamics simulations starting from separated reactants. Boxed molecular dynamics (BXD)^29,30^ and reaction tracking algorithms^31,32^ were used to accelerate bimolecular abstraction in the MD runs and to determine when reaction occurs. The functionality within the ChemDyME^33^ code was used for this.

**Analyzing the distribution of reaction energy between product modes**: Postreaction, the kinetic energy in all 3N normal modes of each product moiety was tracked, and then the virial theorem was applied to obtain the total energy in each mode using the approach described by Glowacki and co-workers.^13^

### 2.1 Potential Energy Surface Fitting

**Figure 1:**
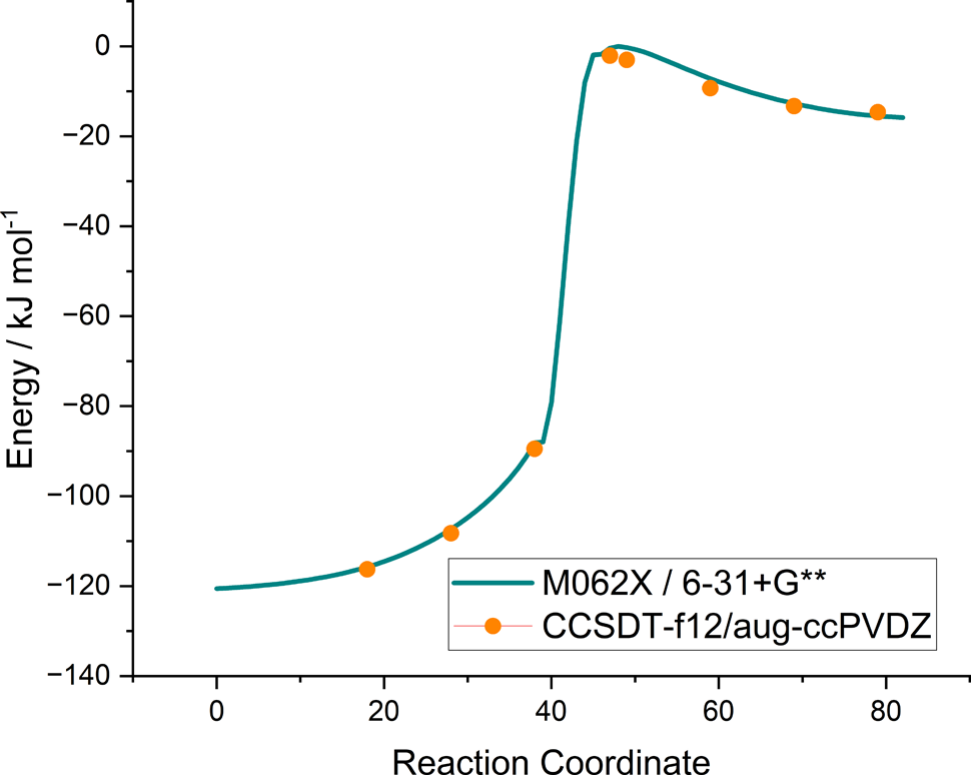
Energies along the intrinsic reaction coordinate (IRC) of the OH + HC(O)C(O)H → HC(O)CO + H_2_O reaction as calculated at the M062X/6–31+G** level of theory. Single point CCSD(T)/aug-cc-pVDZ calculations have been performed at selected geometries along the IRC. All energies are given in kJ mol^–1^.

In this work, a neural net (NN) force field from the PhysNet code was utilized.^27^ This code has the advantage of learning forces in addition to energies, negating the need to generate numerical derivatives of the energy for the MD code, and PhysNet has previously been shown to fit reactive small molecule systems well.^28^ However, to generate the force field from PhysNet, a large amount of *ab initio* data needed to be generated which the NN force field could be fit to. To generate the *ab initio* data, we ultimately settled on the M062X functional with the modest 6–31+G** basis set, as implemented in Gaussian. The M062X functional is extensively parametrized to these small molecule reactions and generally produces very accurate energies and forces for the systems we have previously studied.^5^ Additionally, DFT methods are less obviously dependent on the basis set size. Figure 1 shows the energies along the OH + glyoxal intrinsic reaction coordinate (IRC) at the M062X/6–31+G** level and CCSD(T)-f12/aug-cc-pVDZ level at selected points, showing excellent agreement, well within the uncertainty of the estimated CCSDT calculations (4 kJ mol^–1^).

To sample a greater amount of the configuration space of the OH + glyoxal reaction, the IRC was extended to more configurations in the association and dissociation regions of the reaction. In addition, several extra configurations from low level (PM6) trajectories of the reaction were added. For each point in our extended reaction path, 488 pairwise 0.05 Å displacements of each Cartesian coordinate were generated to sample vibrational configuration space orthogonal to the reaction path. In total, 116591 M062X energies and forces were generated, and the NN was fitted to 90000 of these points with the remaining 26591 points used for validation.

To refine these fits further, we employed a multigenerational approach to fitting the NN PES. After fitting a first generation NN PES to the 90000 points described above, this NN PES was used to generate two reactive trajectories for the OH + glyoxal system. From these trajectories, a further 2000 training data and 1000 validation data were added, and the NN PES was refit producing a second generation NN PES.

**Figure 2:**
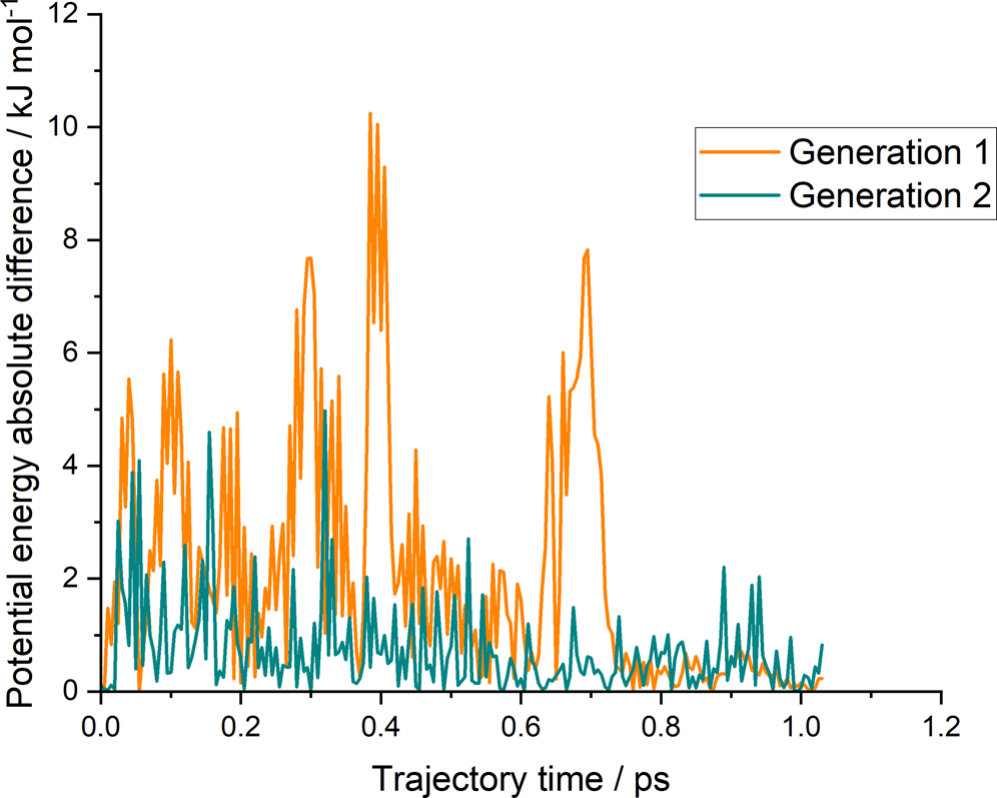
Energies along an OH + glyoxal trajectory propagated with the second generation NN force field. This plot compares the absolute difference between the M062X/6–31+G** reference energy and the energy from the first (orange) and second (cyan) generation NN force fields.

For all NN fitting, the PhysNet code was used in a relatively “black box” manner, and most hyperparameters were chosen to be the same as an example input. The PhysNet input and resulting NN force field can be found in a Zenodo repository.^34^Figure 2 compares the first and second generation NN PESs against M062X/6–31+G** reference energies for geometries from a brand-new reactive trajectory based upon the second generation NN PES. It should be noted that none of the points in this trajectory had been explicitly included in the NN training process. The resulting second generation NN PES was found to predict forces with a maximum root-mean-square deviation (RMSD) of 7.6 kJ mol^–1^ Å^–1^ and a mean RMSD of 2.4 kJ mol^–1^ Å^–1^. The corresponding values for the first generation NN PES are 18.4 and 4.2 kJ mol^–1^ Å^–1^ indicating a substantial improvement in accuracy between generations. Given our estimated error on the M062X energies of 4–8 kJ mol^–1^, it was felt that this second-generation PES was of sufficient accuracy for the present purposes.

### 2.2 Initial Conditions Sampling

All MD trajectories were simulated in the current work using the ChemDyME software.^33^ The MD simulations utilized a classical equation of motion, and all calculations were performed in the microcanonical (constant energy) ensemble. Initial conditions were sampled at a set total energy from a Maxwell–Boltzmann distribution and then equilibrated in one of two ways prior to propagating the molecular dynamics. A time step of 0.5 fs was used. For most of this work, the trajectories were started at the geometry of the transition state as optimized using the NN force field. The breaking and forming bonds were then fixed using the internal Rattle constraints of the ASE code package, and all other internal coordinates were allowed to equilibrate for 50 ps. This approximates a classical thermal sampling of all modes orthogonal to the reaction coordinate. We have named this sampling method “Equilibrated Transition State Sampling,” ETSS. We note that fixing the breaking and forming bonds is only an approximation to the true reaction dividing surface, and this is something we intend to investigate in future work.

The alternate sampling method used in the work started with the OH and glyoxal moieties separated by 10 Å and randomized velocities according to a Maxwell–Boltzmann distribution at a specified temperature. This separation of the moieties was then maintained for 50 ps using a BXD constraint, to equilibrate the system. To accelerate the bimolecular reaction, a BXD constraint was applied along the bond distance between the O atom of the OH reagent and one of the symmetrically indistinguishable hydrogens in glyoxal. Every time this bond length increased, the BXD constraint was applied to force these two atoms into closer proximity. This BXD constraint was enforced until the O–H bond length reached 1.7 at which point the BXD constraint was only enforced if the bond length stretched beyond this 1.7 Å limit. This served to keep the two moieties in the van der Waals region, accelerating the abstraction reaction. This approach to accelerating bimolecular reaction has been described previously.^33^

For the simulations starting from the separated reactants, we also needed techniques to monitor the MD trajectory for reaction. Here we used the Transition State Search using Chemical Dynamics Simulations (TSSCDS) method^31^ as implemented in ChemDyME which checks the trajectory at each frame to observe whether an existing bond is breaking and a new one forming. When this criterion was met, we allowed the trajectory to continue for a further five timesteps to ensure the criterion remained met. We then turned off the BXD constraints, and the trajectory was then run for a further 2 ps to examine the postreaction dynamics. In the case of ETSS, after the 50 ps equilibration, the constraints on the breaking and forming bonds were removed and the trajectory was simply allowed to propagate downhill from the transition state toward products. Regular checks were made to ensure that the trajectory proceeded to form the correct HC(O)CO and water products.

**Figure 3:**
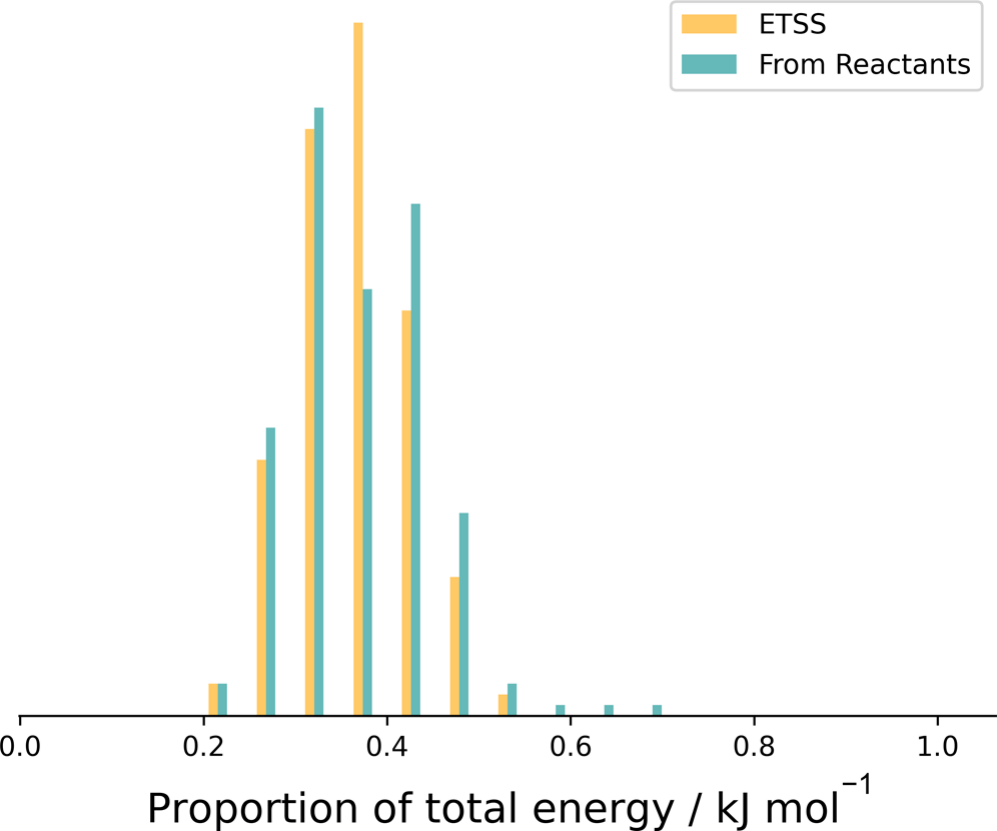
Proportion of the total energy in the HCOCO fragment based on simulations using ETSS (orange) and starting from the separated reactants (cyan) for initial conditions sampling. In both cases, the trajectories are run at a total energy of 50 kJ mol^–1^ above the saddle point energy. Here, only 200 data points are sampled for each case, and the data are histogram into 20 boxes.

To benchmark our saddle point sampling method of generating initial conditions, we have compared trajectories starting from the separated reactants to those starting from the saddle point. These simulations were performed with 50 kJ mol^–1^ energy above the reaction barrier. These results consist of only 200 MD runs for each model, and given the relatively low resolution, a bar chart is used to represent the histogram data rather than the line charts used elsewhere. The resulting product energy distributions are shown in Figure 3 and, broadly speaking, the agreement is good. There are some discrepancies, but these may be due to insufficient sampling in the case of the trajectories starting from the reactants since these simulations are considerably more computationally expensive. We also have not fully investigated whether the BXD constraint might slightly perturb the results from the full trajectories. We intend to investigate this difference more thoroughly over a range of systems in the future, but for the present work, these results indicate that our sampling from the transition state is a reasonable approximation to the full dynamics starting from reactants at substantially reduced computational cost.

**Figure 4:**
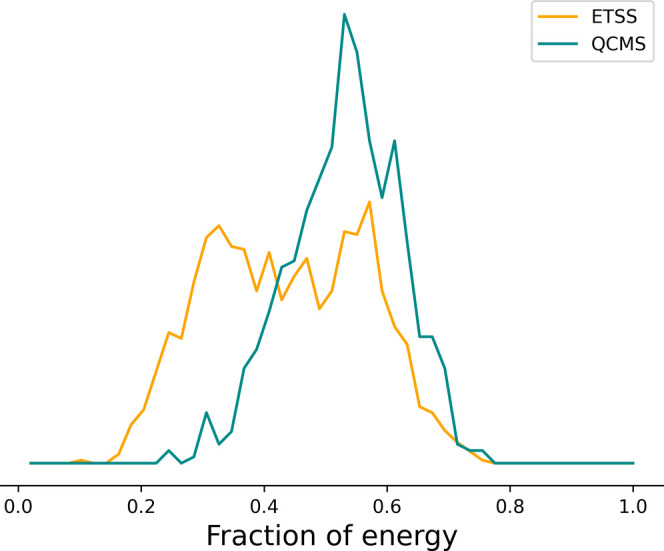
Proportion of the total energy in the HCOCO fragment based on simulations using ETSS (orange) and QCMS (cyan) methods for initial conditions sampling. In both cases, the trajectories are run at a total energy of 110 kJ mol^–1^ above the saddle point energy corresponding to the zero-point energy of the saddle point normal modes.

It is also important to benchmark our new ETSS method against the commonly used quasi-classical microcanonical sampling (QCMS) method for generating initial conditions. This comparison is shown in Figure 4. For this comparison, we have generated initial coordinates and velocities in two ways; from a Gaussian, Born–Oppenheimer molecular dynamics (BOMD) calculation and from our ETSS. The BOMD calculations are performed at 0 K, but once zero-point energy is added, this is equivalent to having 110 kJ mol^–1^ total energy, which is the energy we assigned in our classical ETSS. Both methods of initial conditions sampling were confirmed to give the same total energy in the simulations, and in both sampling cases, the trajectories were propagated using our ChemDyME code. Only the initial velocities and coordinates from the BOMD calculation were read by the ChemDyME code. We emphasize that this comparison is performed using the AM1 semiempirical method, and as such these results are not directly comparable with others in this work. The AM1 method was used since this method was available in the Gaussian code (for the initial conditions selection) and also efficient for use with ChemDyME.

This comparison does highlight additional complexities regarding the use of quasi-classical sampling at lower energies. Glowacki et al.^13^ have previously used both classical and quasi-classical sampling methods for this type of problem and highlighted the necessity of removing the zero-point energies (ZPEs) from the vibrational modes of the products (since the QCMS explicitly adds ZPE to the modes of the transition state). However, in the current work, we have found that even at 50 kJ mol^–1^ excess energy, ZPE conservation is typically violated in at least one of the product modes (i.e., when removing ZPE in a postanalysis of the products, one or more modes end up with negative energy).

**Figure 5:**
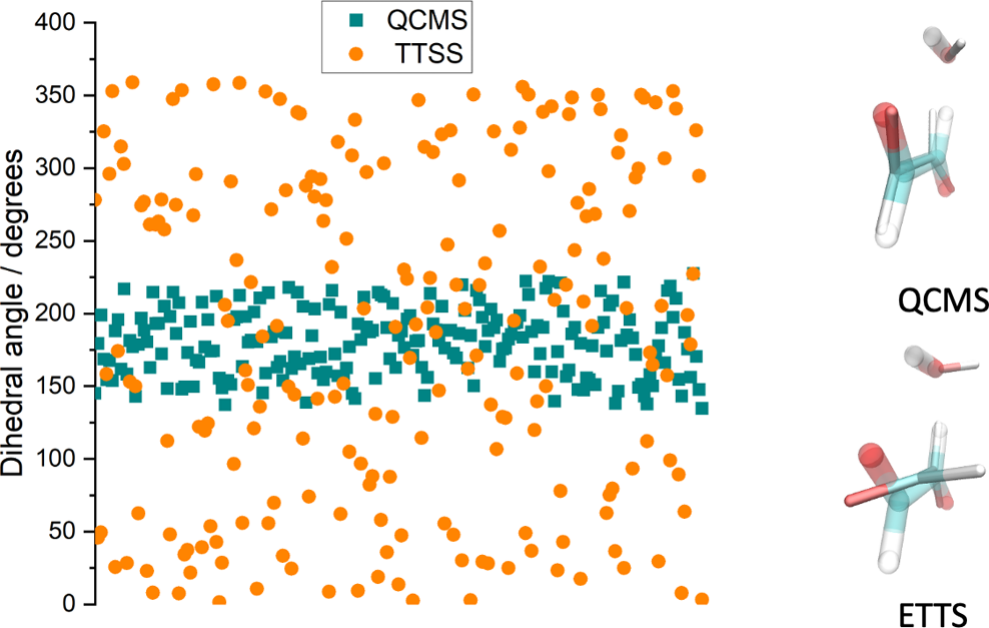
Variation in the C–C–O–H dihedral angle determining the direction of H of the OH moiety relative to the C–C bond in the glyoxal. Both the QCMS (cyan) and ETSS (orange) methods are compared. Also shown are the structures of the maximum deviations in dihedral angle superimposed upon the optimized saddle point structure.

One clue about the difference between ETSS and QCMS is given in Figure 5. This compares the spread of dihedral angles corresponding to the direction of H in the OH moiety of the transition state (formed from the two C atoms, O of OH and H of OH). We also show structures superimposing the maximum deviations of this dihedral angle relative to the reference structure in each case, and it is clear that our ETSS methods sample much more conformational space in this dihedral. This is not surprising. Inherent in the microcanonical method is the assumption that vibrations are harmonic oscillators, and for “floppier” motions like torsions, the assumption is less valid. These results are supported by a detailed previous comparison of the QCMS method against an anharmonic sampling scheme.^35^

### 2.3 Postreaction Energy Distribution

The aim of the work is ultimately to calculate how the energy of an abstraction reaction is distributed between the internal modes of the products and the translational energy. To determine the kinetic energy in each mode of each fragment (including the three translation modes), we used the approach described by Glowacki et al.^13^ as follows. First, it was necessary to project the Cartesian velocities into the normal modes of the fragment:
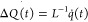
1where *L* is the Hessian matrix and  are the Cartesian velocities. The kinetic energy in the ^th^ mode could then be obtained from
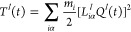
2where the indices *i* and α run over the atoms and the Cartesian x,y,z coordinates, respectively, and *m*_*i*_ is the mass of the *i*th atom.

Determining the potential energy in each mode from trajectory data was more challenging. It is relatively simple in the harmonic limit, but Glowacki and co-workers identified that getting accurate mode potential energies in real anharmonic modes is problematic. Considering this, in the current work, we followed the approach of Glowacki et al.^13^ in utilizing the virial theorem to get the total energy in each mode.
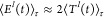
3

Here, τ is some time window over which the kinetic energies  are averaged. In this work, a time window of 3 ps was found to be sufficient for convergence, having first let the reaction relax for 1 ps postreaction.

### 2.4 MESMER Calculations

**Figure 6:**
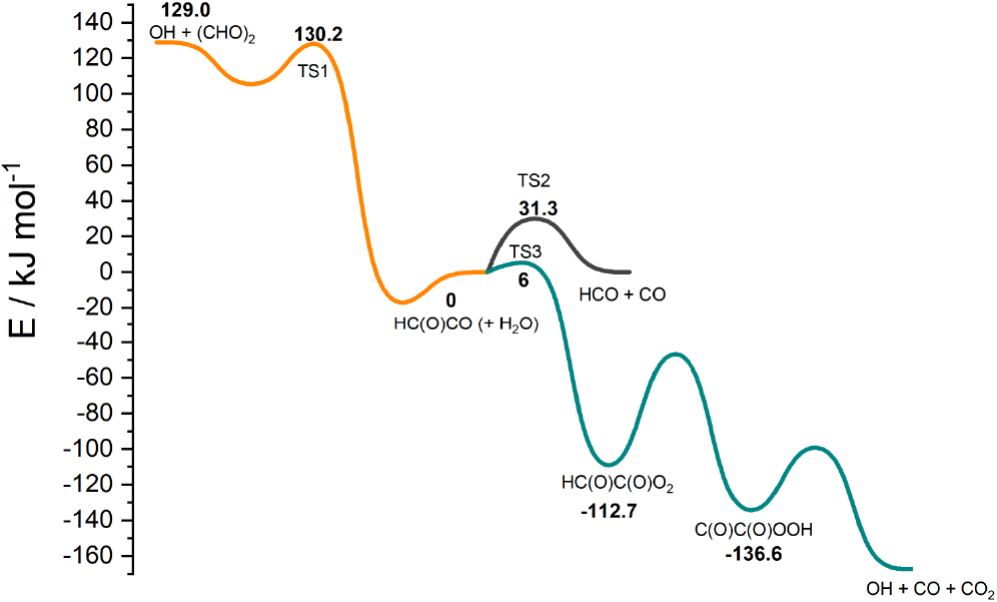
Stationary points on the OH + glyoxal potential energy surface, as used in our MESMER calculations. This figure uses energy values from ref (5).

To benchmark our trajectory results, we inputted the energy product distributions calculated dynamically into a master equation simulation to compare with experimental results for the OH + glyoxal + O_2_ system. These master equation simulations were performed using the MESMER open-source software,^36^ and the input used for the master equation simulation was for the most part identical to that used in our previous work.^5^ The stationary points of the OH + glyoxal + O_2_ system were characterized by previous calculations at the CCSD(T)-f12/aug-cc-pVTZ//M062*X*/6–311+G** level. The master equation also included explicit treatment of all internal rotational modes as 1D hindered rotations. Energy transfer was treated using an exponential down model, and all simulations were performed with an energy grain size of 100 cm^–1^. The potential energy surface is shown in Figure 6 and shows the stationary points of the potential. We note that multireference calculations in our previous work showed a barrier of ∼6 kJ mol^–1^ along the path of association of O_2_ with HCOCO (TS3). These calculations are far from conclusive; however, we find a much-improved master equation fit to the experimental data if we include TS3. This O_2_ addition is highly correlated with the dissociation channel controlled by TS2, and a smaller fitted barrier for TS2 would be consistent with a barrierless O_2_ addition. Within the errors of the MD calculation and the *ab initio* calculations, it is not possible to say anything more conclusive regarding this channel in the present work.

The main change in this work compared to the previous master equation simulations^5^ is that we have replaced a statistical prior distribution for working out the energy partitioning of the OH + glyoxal reaction energy into HCOCO and water, with a dynamical distribution taken from the trajectory calculations.

For this work, we chose an asymmetric Gaussian equation to fit the dynamical distributions:
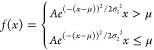

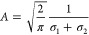
4where μ corresponds to the peak of the distribution, σ_1_ corresponds to the right-hand standard distribution, and σ_2_ corresponds to the left-hand standard distribution.

## 3 Results

### 3.1 Molecular Dynamics Distributions

**Figure 7:**
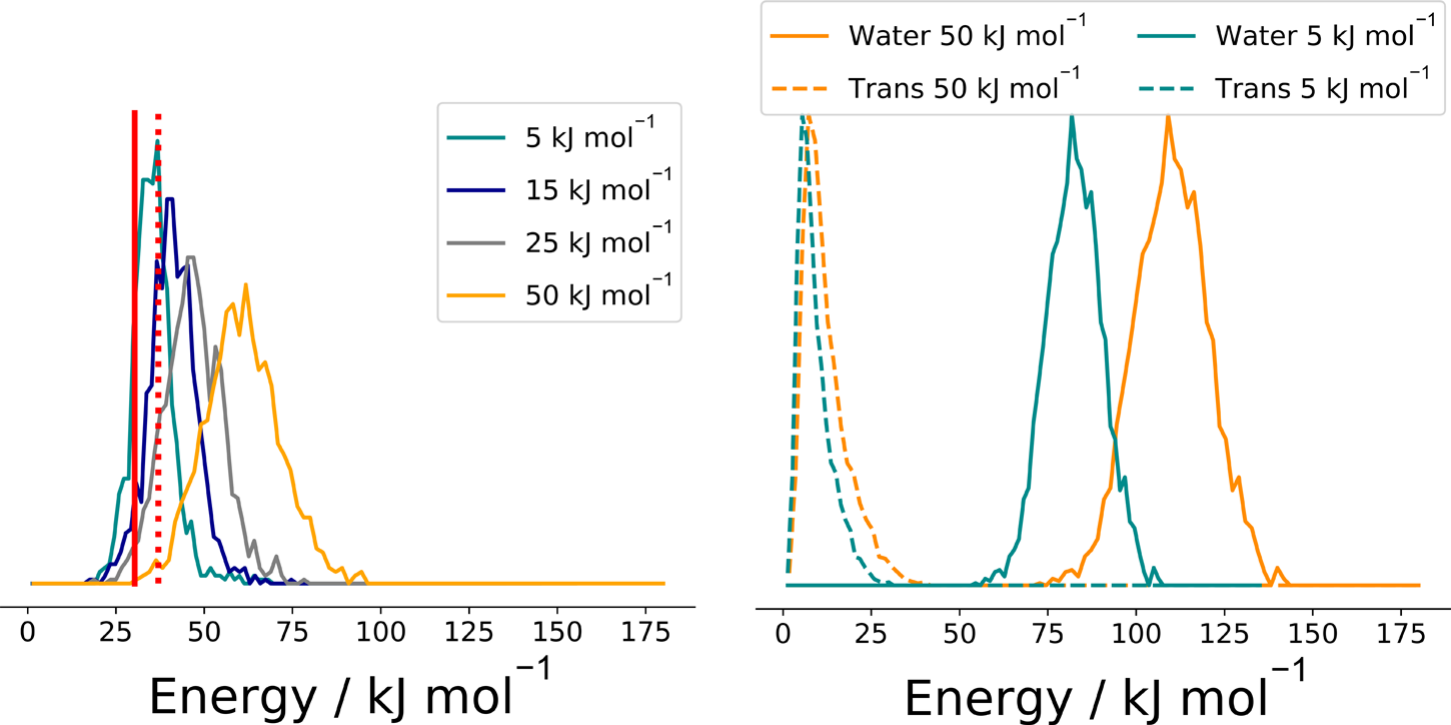
Left panel: calculated total energy deposited in the HC(O)CO fragment from the OH + glyoxal reaction at 5 (cyan), 15 (blue), 25 (gray), and 50 (orange) kJ mol^–1^ above the saddle point. Also shown are red vertical lines showing the energy of the dissociation barrier to form HCO + CO for both the *ab initio* value of the barrier (solid line) and the fitted value of the barrier presented in this work (dotted line). It should be noted that the portion of the HC(O)CO distributions above the relevant dissociation threshold promptly decompose. Right panel: water (solid line) and translation energy (dashed line) distributions at 50 kJ mol^–1^ (orange) and 5 (cyan) kJ mol^–1^.

Utilizing the above framework, we have performed MD simulations at four different energies between 5 and 50 kJ mol^–1^ above the electronic energy of the saddle point for the OH + glyoxal reaction. For reference, the Boltzmann population of energies above the reaction saddle point is shown in Figure S1 based on master equation calculations, and at 298 K, the peak of this distribution is located at ∼20 kJ mol^–1^. From these MD simulations, it was possible to histogram the proportion of the total reaction energy which goes into the different modes of HC(O)CO and water products. Figure 7 shows the normalized energy distributions of the HC(O)CO fragment for simulations at different energies above the saddle point. As the total available energy increases, the energy in the HC(O)CO fragment also increases, and it is also noticed that the Gaussian-like profile of the HC(O)CO energy distribution becomes more asymmetric and skews to the left as the energy increases. The right panel of Figure 7 shows the corresponding distributions of energy in the water and translational modes for the 5 and 50 kJ mol^–1^ simulations, and it is clear that the majority of the reaction exothermicity goes into the water moiety, despite having a lower state density than the HC(O)CO cofragment. This supports previous observations that the energy partitioning between products is highly nonstatistical. We have tested the new statistical models proposed by Danilack et al.^21^ on the current system and get values between 0.53 and 0.73 for the mean fraction of energy in the HC(O)CO fragment, substantially more than predicted by the dynamics simulations performed here where the majority of the reaction energy goes into the water modes. Figures S2 and S3 show more detailed results from the dynamics simulations, exploring the time dependence of the energy distribution among modes.

### 3.2 Master Equation Analysis

As mentioned above, the OH + glyoxal (+O_2_) system is a very sensitive test of the product energy distributions. The full scheme is as follows:
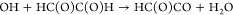
R1
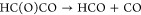
R2
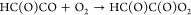
R3
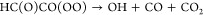
R4

**Figure 8:**
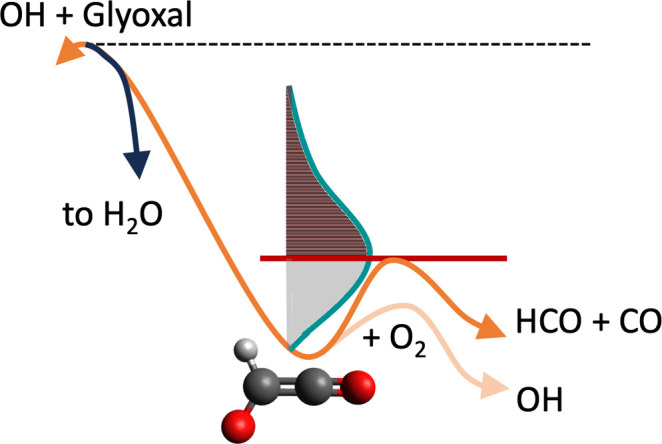
Schematic of the salient features of the OH/glyoxal/O_2_ system. The key HC(O)CO species is formed from the OH + glyoxal abstraction reaction. A total of 130 kJ mol^–1^ excess energy (plus thermal energy contributions) is then available to be deposited between the water and HC(O)CO fragments. A representative distribution of energies in HC(O)CO is shown. The fraction of HC(O)CO formed at energies above the 31.3 kJ mol^–1^ dissociation barrier (shaded red) virtually all undergoes prompt decomposition. The lower energy fraction (shaded gray) undergoes a competition between thermal decomposition and reaction with O_2_ to recycle OH radicals.

In the context of the current work, the key to this system is the competition between dissociation of the HC(O)CO (R2) radical formed from OH + glyoxal (R1) and reaction of HC(O)CO with O_2_ which ultimately recycles OH (R3 and R4).^14^Figure 8 shows these competing processes schematically. In Figure 8, the black dashed line indicates the total reaction exothermicity of the OH + glyoxal reaction that is available for distribution between HC(O)CO and water (130 kJ mol^–1^) and that there will be additional thermal energy. A representative distribution of energies within HC(O)CO is shown, and shading indicates the portion of the distribution at energies in excess of the HC(O)CO barrier height (31.3 kJ mol^–1^) in brown. This brown shaded portion of the distribution is found to dissociate to HCO + CO rapidly (see Figure S6) while the gray portion of the distribution undergoes greater competition between bimolecular reaction with O_2_ to form OH radicals and thermal decomposition of HC(O)CO. The key observation is that in the limit of high [O_2_], thermal decomposition of HC(O)CO becomes uncompetitive with bimolecular reaction of HC(O)CO with O_2_ and as such almost all HC(O)CO that does dissociate, does so via the prompt mechanism, i.e., comes from the red portion of the distribution. Previous experimental work generated yields for OH recycling (via R4) in the OH + glyoxal reaction as functions of pressure and [O_2_]. At 212 K, in the limit of high [O_2_], the OH recycling yield tends to an asymptotic value of ∼0.36 indicating that 64% of HC(O)CO formed from the OH + glyoxal reaction decomposes promptly and cannot be intercepted. At lower values of [O_2_], the OH recycling yield decreases with [O_2_] indicative of the competition between thermal decomposition of HC(O)CO and the bimolecular [O_2_] reaction which increasingly favors decomposition as the [O_2_] is decreased.

In previous master equation modeling, we used a statistical, prior distribution model to generate the energy distribution in the HC(O)CO fragment following reaction R1.^5^ However, with this statistical model, we found our master equation model predicted far too much prompt HC(O)CO dissociation, and the prior distribution model had to be modified to partition substantially more energy into the water. Additionally, it was found in our previous modeling^5^ that the prior distribution had to be modified differently to fit the experimental data at each different temperature, with the best fits occurring with a slight increase in the fraction of energy channeled into HC(O)CO with increasing temperature. In this work, we have found an improved fit using a modified prior distribution. This new fit is compared with the MD fits in Section S2; however, the fact remains that a prior model is only suitable for modeling this reaction when modified in a somewhat *ad hoc* manner.

In this work, we parametrized the MD distributions in Figure 7 as asymmetric Gaussian curves (see Section S2), and we refitted the experimental data incorporating these dynamical distributions into the master equation. We fitted the available experimental OH yields^5^ by varying the energy transfer parameters of the HC(O)CO relaxation by helium bath gas (Δ*E*_down,295 K_; its temperature exponent, (Δ*E*_down_ = Δ*E*_down,295K_ × (*T*/295)*^n^*) is fixed to 1), the barrier height to HC(O)CO decomposition, and the inverse Laplace transform *A* factor (*A*_ILT_) for the HCOCO + O_2_ reaction using the built in Levenberg–Marquardt algorithm in MESMER.^37^ The fitted results are given in Table 1 compared to the previous work.^5^

**Table 1:** Fitted Parameters from the MESMER Fits to the Experimental Data from Lockhart et al.^14^^a^

	this work	ref (5)
TS2 (kJ mol^–1^)	37.4 ± 0.1^b^	31.3
Δ*E*_down,295K_ (cm^–1^)	84.5 ± 10.0	388
*A*_ILT_	(3.4 ± 0.7) × 10^–13^	1.7 × 10^–12^

a All errors are at the 2σ level. Also shown is a column with the corresponding values for the master equation calculations performed in ref (5).

b The 2σ uncertainties here only incorporate the statistical errors from the MESMER fits and do not reflect any in the dynamical distribution.

**Figure 9:**
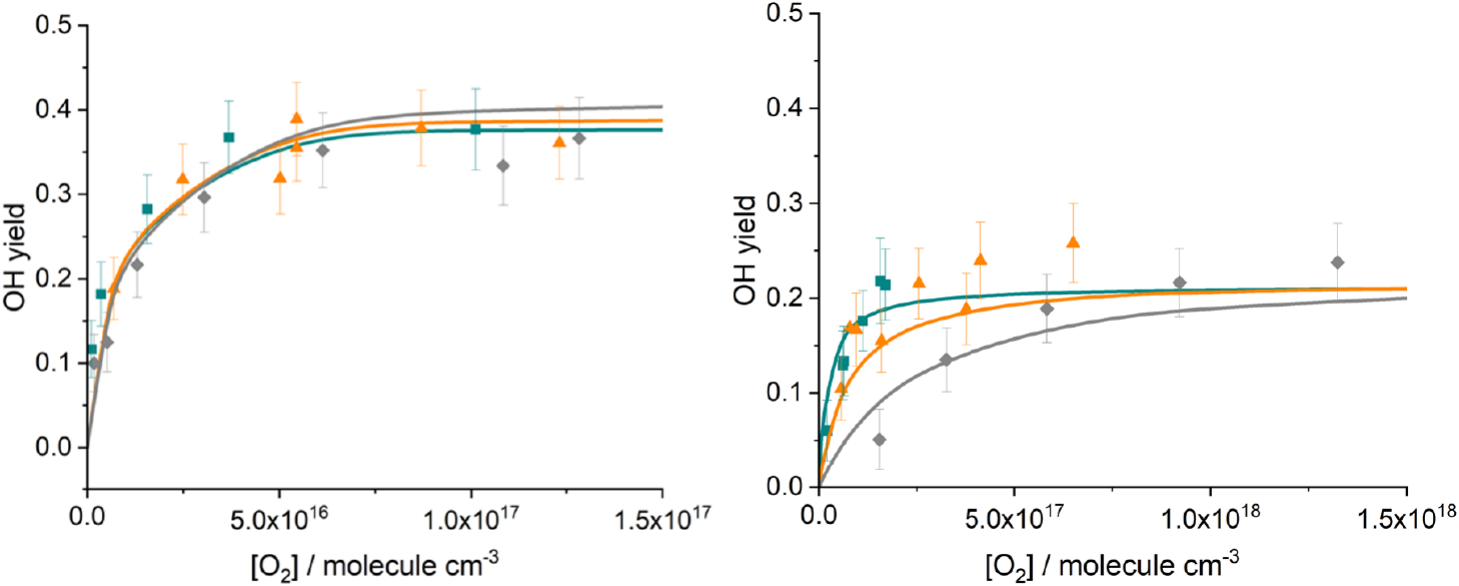
OH yields from master equation simulations for OH + (CHO)_2_ + O_2_. These master equation simulations include the nonthermal postreaction distribution in the HC(O)CO radical from the MD simulations described herein. The points in these figures correspond to experimental data from ref (14), and the lines correspond to the MESMER results. Three pressures for the He bath gas are shown, 80 Torr (gray). 20 Torr (orange), and 5 Torr (cyan). The left panel is at 298 K while the right panel is at 212 K.

**Figure 10:**
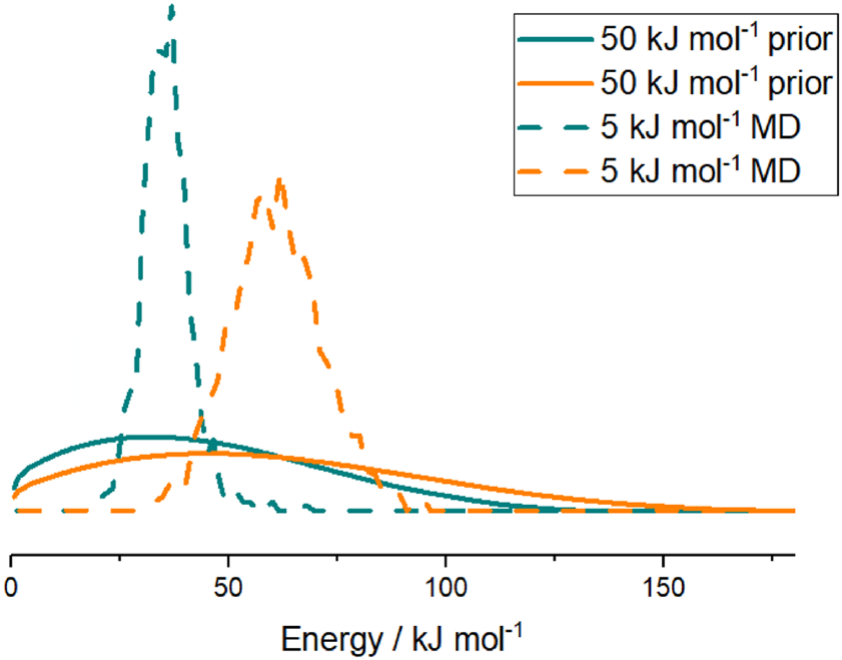
Comparison of dynamical HC(O)CO energy distributions (dashed lines) produced in this work with those from a modified prior distribution (solid lines) used in previous master equation modeling in ref (5) These distributions are shown at 5 kJ mol^–1^ (cyan) and 50 kJ mol^–1^ (orange) above the barrier.

A comparison between the fitted MESMER simulation and theory is shown in Figure 9 for 212 and 298 K. These plots show that, without modification, our MD-based energy distribution gives excellent fits to the experimental data with only a relatively modest change of 5.9 kJ mol^–1^ to the *ab initio* barrier for HC(O)CO decomposition. Furthermore, we find that using the MD distributions we predict a significant temperature dependence on the high [O_2_] limiting OH yields. Figure 10 compares the MD distributions produced here with the modified prior distribution used in ref (5). The prior distributions are seen to be far broader than their MD counterparts and appear significantly less sensitive to the energy above the reaction saddle point. The energy dependence of the MD distributions gives rise to the different limiting values of the OH yield at different temperatures as seen in Figure 9. At 212 K, a greater proportion of the HC(O)CO radical is formed below the dissociation threshold and, thus, in the limit of high [O_2_], a larger fraction of HC(O)CO may be intercepted by O_2_ to form OH (an OH yield of 0.4 vs 0.2 at 298 K). Compared to the previous modeling, the improved fit quality can also be observed in the pressure dependencies of the OH yields with our MD-based modeling correctly reproducing the pressure dependence of the 298 K data due to falloff effects in the thermal HC(O)CO decomposition process and lack of pressure dependence at 212 K where the HC(O)CO decomposition is closer to its high-pressure limit.

Undoubtedly, there are sources of error in our dynamical distributions, chiefly coming from the lack of consideration of quantum effects and errors associated with fitting the distributions. Given that the HC(O)CO energy distribution and the corresponding HC(O)CO decomposition are highly coupled, the 5.9 kJ mol^–1^ difference between *ab initio* value and the fitted barrier is well within the combined uncertainty of the *ab initio* calculations (∼4–8 kJ mol^–1^) and the MD calculations. In the Supporting Information (Section S2), we explore how the fit parameters change if we modify our dynamical distributions to give a more realistic, though albeit qualitative, sense of the uncertainties in the three fitted parameters.

## 4 Conclusions

This work uses a simple, generalizable, MD-based methodology to predictively model postreaction energy partitioning in the OH/glyoxal/O_2_ system. We find that with modest changes to the barrier height, our HC(O)CO energy distribution calculated via MD can quantitatively reproduce the observed experimental data across the full experimental temperature range. Compared to previous work,^5^ this model can be used *a priori,* without modifying the distributions to fit experiment, and we suggest that this new approach forms an important theoretical tool for predictive modeling of activated systems. A key part of our approach is that the efficient NN PES allows for greater sampling of initial conditions in the transition state region. Compared to the commonly used QCMS scheme, results from our ETSS approach show product energy distributions which are shifted toward lower energies. Given the stringent benchmark that OH/glyoxal/O_2_ presents, the results presented herein suggest that our classical ETSS approach can accurately predict postreaction energy distributions for these types of systems.

There is a growing body of high-profile papers,^3,5,6,9,10^ highlighting the importance of the chemical activation effects considered here, and the framework used herein is readily extendable to other systems. Clearly, the H_2_O fragment considered in this work is formed with far higher energies than its HC(O)CO cofragments, and it will be informative to see the extent to which this “hot” water trend persists to other systems. With more data, it may also be possible to make a simpler empirical model that more accurately reflects the true postreaction dynamics. For this system, we find that the recently proposed statistical models of Danilack and Goldsmith overestimate the amount of energy in the HC(O)CO fragment, and further work across a range of smaller systems is required to explore this. We note that approaches based upon the “sudden vector projection” approach of Guo and Jiang^38^ look promising, and we intend to explore this in more detail. In future work, it would also be informative to introduce quantum effects into the dynamics. The commonly used ring polymer molecular dynamics method^39^ is not applicable to this problem since this necessitates heavily thermostated dynamics; however, approaches based upon coupled coherent state type may prove fruitful.^40^

## Supporting Information Available

Word document containing additional MD results and the full master equation input file used (PDF)
