# Supplementary material for: Solving the OH + Glyoxal Problem: A Complete Theoretical Description of Post-Transition-State Energy Deposition in Activated Systems
Source: J Phys Chem A. 2024 Feb 20;128(8):1501–10. doi: 10.1021/acs.jpca.3c07823 (PMC10910583; doi:10.1021/acs.jpca.3c07823)
Supplement: Supplementary file 1 — jp3c07823_si_001.pdf [file jp3c07823_si_001.pdf]

Supporting Information
Solving the OH + Glyoxal problem: A Complete Theoretical Description of Post Transition State Energy Deposition in Activated Systems.

Robin Shannon*[1], Mark A. Blitz[1,2] and Paul W. Seakins[1]
1 – School of Chemistry, University of Leeds, Leeds, LS2 9JT, UK
2 – National Centre for Atmospheric Science, University of Leeds, Leeds, LS2 9JT, UK.

S1. Extended MD results.

The section presents some extended MD results. Firstly, Figure S1 shows Boltzmann populations above the OH + glyoxal abstraction saddle point (TS1) at 298 K and 212K from the MESMER simulations of the system. This plot is provided to aid the reader in converting from the microcanonical or energy resolved regime using in the dynamics simulations to a canonical or temperature resolved picture.

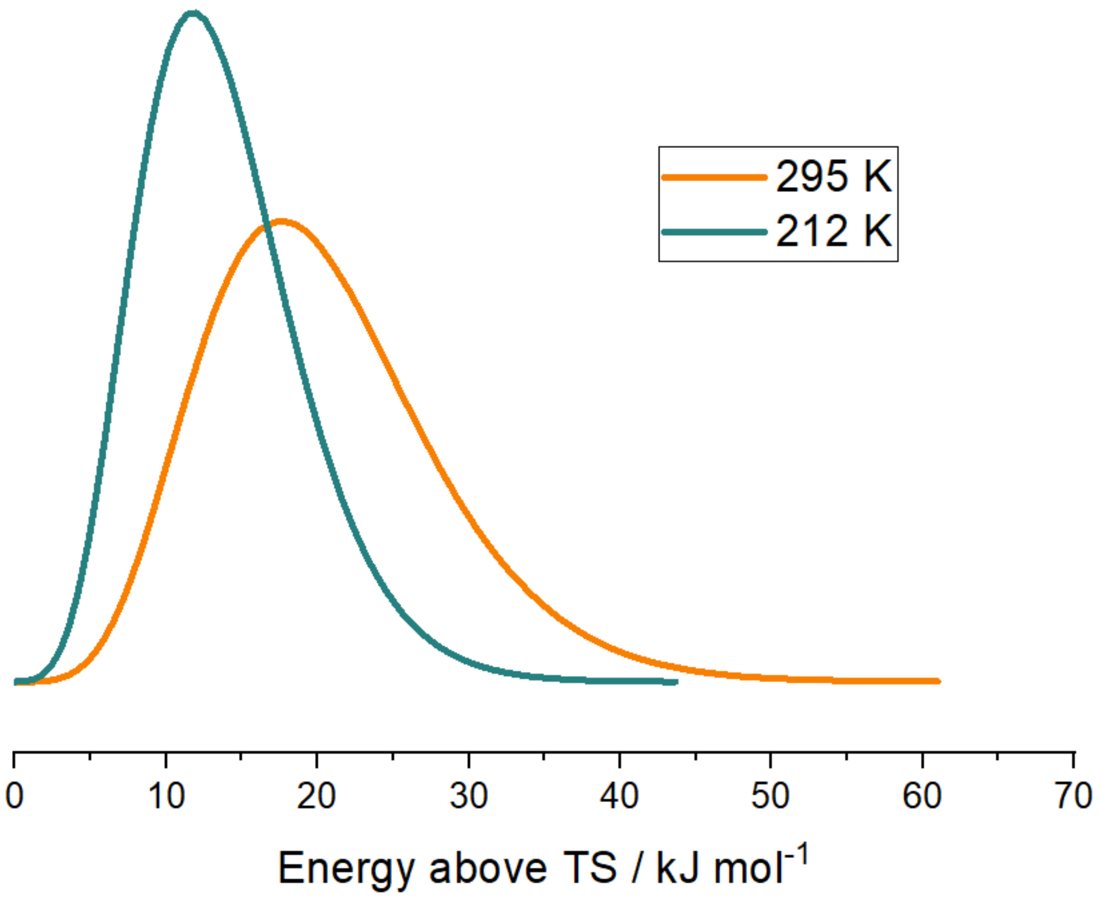

Figure S1: Normalised Boltzmann distribution of energies above the OH + glyoxal abstraction TS at 298K (cyan) and 212K (orange) as calculated by MESMER.

Figures S2 and S3 how the kinetic energy in different modes evolves in time as reaction occurs. Figure S2 shows the energy split between translational, rotational and vibrational components and Figure S3 shows the 3 vibrational modes of the water. Interestingly in both cases the kinetic energies reach an equilibrium between 0.5 and 1 picosecond post reaction. With regards to the water modes the energy is relatively evenly split but interestingly it is the bending motion which gain the largest proportion of the reaction exothermicity

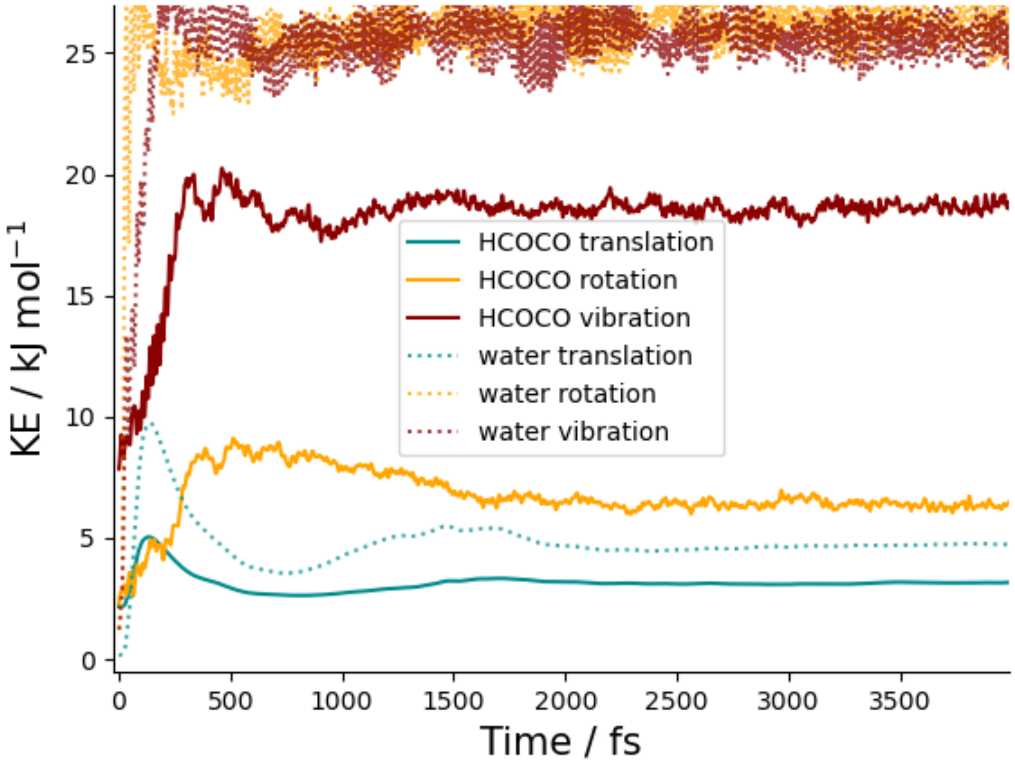

Figure S2: Kinetic energy as a function of time from simulations at 50 kJ mol^{-1} total energy. These profiles are averaged over 1000 simulations. The time starts when reaction criterion is triggered. Here we show how kinetic energy is split among the mode types of the water and HC(O)CO fragments.

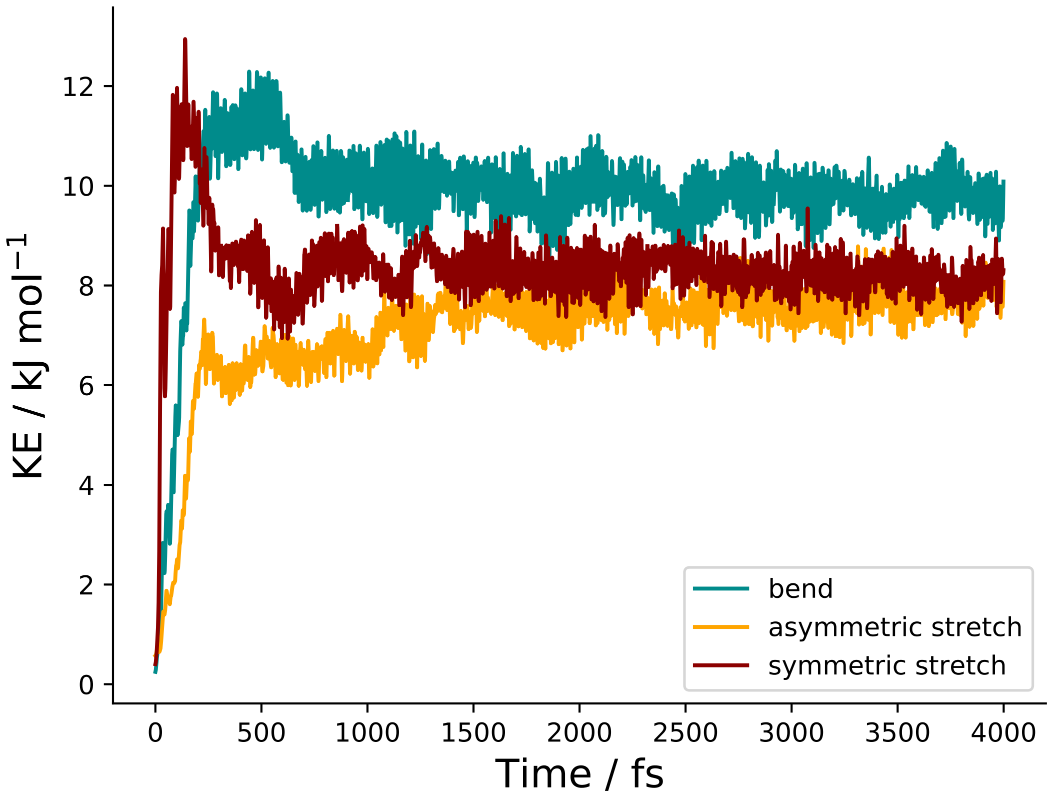

Figure S3: Kinetic energy as a function of time from simulations at 50 kJ mol^{-1} total energy. These profiles are averaged over 1000 simulations. Here we show how kinetic energy is split among three vibrational modes of the water.

S2. Fitting asymmetric gaussian curves to MD distributions

Figure S4 show these fitted parameters as a function of energy and Table S1 shows the gradients and intercepts resulting from linear fits to these parameters. In the future we aim to explore alternative fitting functions and wider energy ranges for a variety of systems to gain a better understanding of these distributions. For the current work we have assumed a linear dependence upon each gaussian parameter with energy although as noted above, there is substantial uncertainty regarding this.

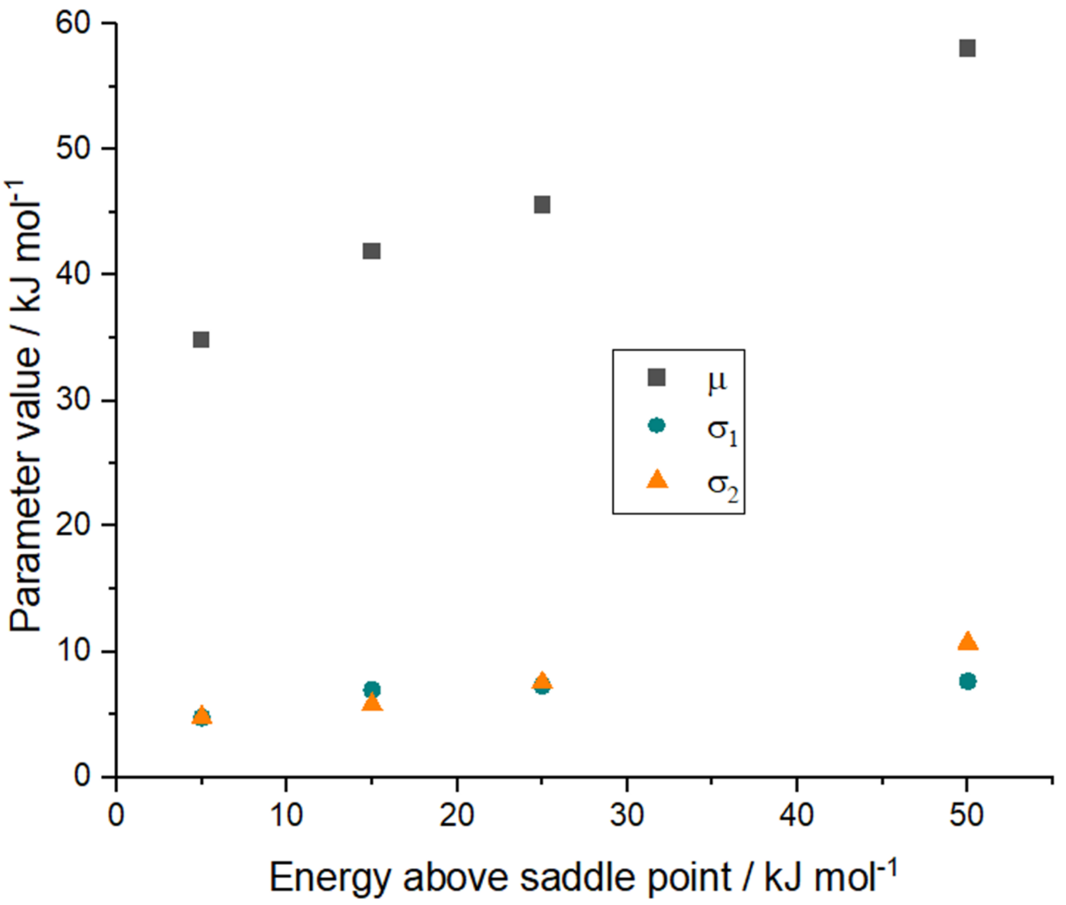

Figure S3: Fit parameters μ (grey), σ_1 (cyan), σ_2 (orange) of Eq. 4 as a function of energy above the saddle point.

	μ	σ_1	σ_2
gradient	0.0059 ± 0.004	0.0006 ± 0.0003	0.0016 ± 0.001
intercept	-34.9 ± 0.8	-1.6 ± 0.3	-13.4 ± 0.4

Table S1: Gradients and intercepts of linear fits to the asymmetric Gaussian parameters in Figure S9. Errors are quoted at the 2σ level.

	Model 1	Model 2 (*a priori*)	Model 3	Modified Prior
TS2 / kJ mol^{-1}	35.5 ± 0.2	37.3 ± 0.2	38.5 ± 0.2	35.1 ± 1.2
$\Delta E_{down,295\ K}$ / cm^{-1}	74.0 ± 16.1	98.3 ± 21.3	139.3 ± 36.6	107.5 ± 46.8

A_{ILT} / molecule^{-1} cm^3 s^{-1}	(3.2 ± 0.7) × 10^{-13}	(3.1 ± 0.6) × 10^{-13}	(3.9 ± 0.9) × 10^{-13}	(4.1 ± 1.4) × 10^{-13}
Modified Prior Order	-	-	-	0.27 ± 0.03
χ^2 per point	1.1	1.1	1.1	1.2

Table S2: MESMER fit results for four different models. Models 1 to 3 use the MD distributions. Model 1 has the μ intercept parameter reduced by 2 kJ mol^{-1}, Model 2 uses the *a prior* dynamics model and Model 3 has the μ intercept parameter increased by 2 kJ mol^{-1}. The final column shows new fit results from a modified prior distribution.

In order to explore the correlation between the fit parameters and dynamical distributions we have performed additional master equation fits with modified dynamical distributions. The dynamical results have been modified by shifting the distributions (the intercept of the μ parameter) 2 kJ mol^{-1} towards both lower (model 1) and higher (model 3) energies. Table S2 compares master equation fits for models 1 and 3 alongside the master equation fits from the unmodified dynamical distributions (model 2). In this table it can be seen from the χ^2 parameter that the fits are of almost identical quality, however despite very small statistical errors for any given fit, the fitted barrier heights for TS2 display an almost linear correlation with the 2 kJ mol^{-1} energy shifts in the modified distributions.

We have also performed new fits with a modified prior distribution and in this case found an almost equivalent fit, significantly improving our previous attempts at modelling this system with a modified prior model. The fact that this fit wasn't found in our previous work[1] highlights the correlations between fitting parameters in this system and demonstrates how sensitive the fit results are to the initial parameter guesses. This fit is included in Table S2 and is similar in quality and parameter values to the 3 MD based fits. Given the complexity of the fits, we make no claim that we have found the true global minimum for the system but since the aim of this work is predominantly to benchmark our new MD approach, we have not explored these master equation fits further.

To compare the new prior distribution fits with our MD model, both are plotted against the experimental data in Figure S5. Here it can be seen that consistent with previous work the modified prior model does not predict any significant temperature dependence in the high [O_2] limiting OH yields. However, a compromise fit is found where the 298 K data is modelled as being further from reaching it high [O_2] limiting rate coefficient. This behaviour is promoted by both the lower ΔE_{down} and A_{ILT} (combined with the 6 kJ mol^{-1} energy for the ILT used here) values compared to the modified prior fits in our previous work. Within the experimental error bars the new modified prior fit is of equivalent quality to our MD results although visually the two models are distinct.

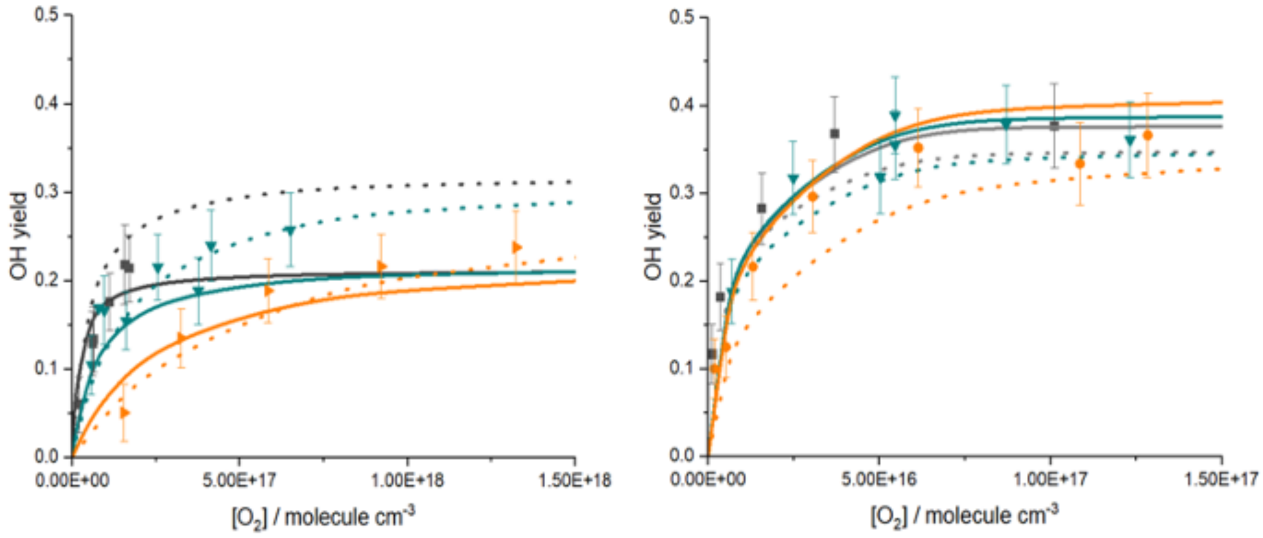

Figure S5: OH yields from Master equation simulations for the OH + $(CHO)_2$ + O_2 reaction system. The points in these figures correspond to experimental data from Ref 1, the solid lines correspond to the MD distributions and the dotted lines correspond to the modified prior distribution. Three pressures are the He bath gas are shown, 80 Torr (grey). 20 Torr (cyan), 5 Torr (orange). The left panel is at 298 K whilst the right panel is at 212K.

Figure S6 shows the microcanonical rate coefficients for dissociation of the HC(O)CO radical as a function of energy. This demonstrates that above the dissociation barrier, the rate coefficients increase extremely rapidly, supporting the observation that almost all HC(O)CO formed above the barrier decomposes instantaneously on the timescale of other processes such as stabilisation or reaction with O_2.

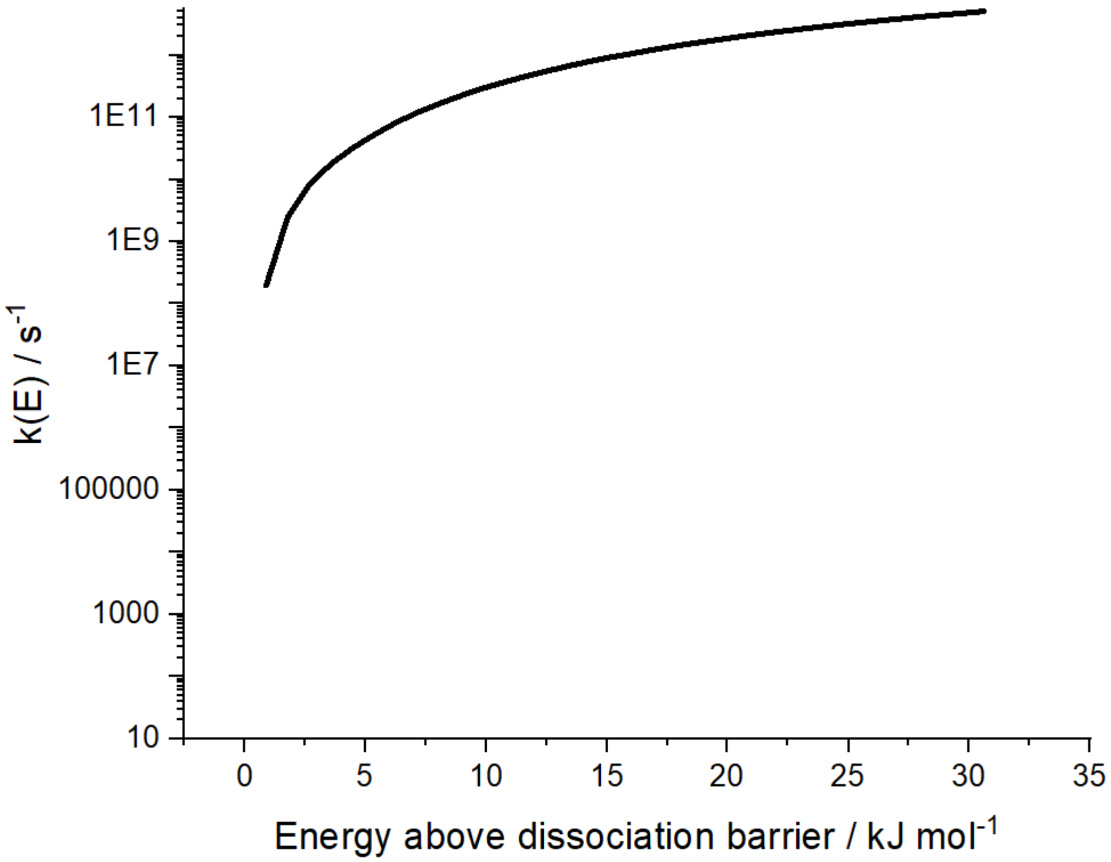

Figure S6: Microcanonical rate coefficients for dissociation of HC(O)CO.

S3. MESMER input

```
<?xml version="1.0" encoding="utf-8"?>
<?xml-stylesheet type='text/xsl' href='../../mesmer1.xsl' media='screen'?>
<me:mesmer xmlns="http://www.xml-cml.org/schema"
      xmlns:me="http://www.chem.leeds.ac.uk/mesmer"
      xmlns:xsi="http://www.w3.org/2001/XMLSchema-instance">
 <me:title> Glyoxal</me:title>
 <moleculeList>

  <molecule id="OH_i">
   <atomArray>
    <atom id="a1" elementType="O" />
    <atom id="a2" elementType="H" />
   </atomArray>
   <bondArray>
    <bond atomRefs2="a1 a2" order="1" />
   </bondArray>
   <propertyList>
    <property dictRef="me:ZPE">
```

```
        <scalar units="kJ/mol">0.0</scalar>
      </property>
      <property dictRef="me:rotConsts">
        <array units="cm-1">19.2438</array>
      </property>
      <property dictRef="me:symmetryNumber">
        <scalar>1</scalar>
      </property>
      <property dictRef="me:vibFreqs">
        <array units="cm-1">3722.0</array>
      </property>
      <property dictRef="me:MW">
        <scalar units="amu">17</scalar>
      </property>
      <property dictRef="me:spinMultiplicity">
        <scalar>2</scalar>
      </property>
      <property dictRef="me:electronicExcitation">
        <array units="cm-1">139.7</array>
      </property>
    </propertyList>
    <me:DOSCMethod xsi:type="ClassicalRotors"/>
  </molecule>

  <molecule xmlns="http://www.xml-cml.org/schema" id="Glyoxal">
    <atomArray>
      <atom id="a1" elementType="C" x3="-0.344494" y3="0.688639"
z3="0.000000"/>
      <atom id="a2" elementType="H" x3="-1.412709" y3="0.750417"
z3="0.000000"/>
      <atom id="a3" elementType="C" x3="0.344494" y3="-0.688639"
z3="0.000000"/>
      <atom id="a4" elementType="H" x3="1.412709" y3="-0.750417"
z3="0.000000"/>
      <atom id="a5" elementType="O" x3="-0.344494" y3="-1.741667"
z3="0.000000"/>
      <atom id="a6" elementType="O" x3="0.344494" y3="1.741667"
z3="0.000000"/>
    </atomArray>
    <bondArray>
      <bond atomRefs2="a1 a2" order="1"/>
      <bond atomRefs2="a1 a3" order="1"/>
      <bond atomRefs2="a1 a6" order="2"/>
      <bond atomRefs2="a3 a4" order="1"/>
      <bond atomRefs2="a3 a5" order="2"/>
    </bondArray>
    <propertyList>
```

```
        <property title="program">
          <scalar>Gaussian 09, Revision D.01</scalar>
        </property>
        <property title="basis">
          <scalar>6-311+G(3df,2pd) (5D, 7F)</scalar>
        </property>
        <property title="Vibrational Frequencies" dictRef="me:vibFreqs">
          <array units="cm-1">137.16 342.01 566.10 822.58 1091.37 1094.26 1346.09 1389.94 1870.76 1883.02 2997.95 3000.79 </array>
        </property>
        <property title="Energy" dictRef="me:ZPE">
          <scalar units="kJ/mol">129.0</scalar>
        </property>
        <property title="Rotational Constants" dictRef="me:rotConsts">
          <array units="cm-1">1.864 0.162 0.149 </array>
        </property>
        <property title="Symmetry Number" dictRef="me:symmetryNumber">
          <scalar>2 </scalar>
        </property>
      </propertyList>
    </molecule>

    <molecule xmlns="http://www.xml-cml.org/schema" id="C1" spinMultiplicity="2">
      <atomArray>
        <atom id="a1" elementType="C" spinMultiplicity="3" x3="0.202227" y3="0.619571" z3="-0.001308"/>
        <atom id="a2" elementType="H" x3="-2.543369" y3="-0.875411" z3="0.811111"/>
        <atom id="a3" elementType="C" x3="0.892783" y3="-0.594844" z3="-0.023778"/>
        <atom id="a4" elementType="H" x3="0.260186" y3="-1.480395" z3="-0.074860"/>
        <atom id="a5" elementType="O" x3="2.135271" y3="-0.577513" z3="0.011170"/>
        <atom id="a6" elementType="O" spinMultiplicity="2" x3="-0.252284" y3="1.670770" z3="0.015351"/>
        <atom id="a7" elementType="O" x3="-2.079583" y3="-0.771427" z3="-0.022468"/>
        <atom id="a8" elementType="H" x3="-2.714112" y3="-0.367193" z3="-0.618167"/>
      </atomArray>
      <bondArray>
        <bond atomRefs2="a8 a7" order="1"/>
        <bond atomRefs2="a4 a3" order="1"/>
        <bond atomRefs2="a3 a1" order="1"/>
        <bond atomRefs2="a3 a5" order="2"/>
        <bond atomRefs2="a7 a2" order="1"/>
```

<bond atomRefs2="a1 a6" order="1"/>
</bondArray>
<propertyList>
<property title="program">
<scalar>Gaussian 09, Revision D.01</scalar>
</property>
<property title="basis">
<scalar>6-311+G(3df,2pd) (5D, 7F)</scalar>
</property>
<property title="File Format">
<scalar>g03</scalar>
</property>
<property title="Energy" dictRef="me:ZPE">
<scalar units="kJ/mol" >104.5</scalar>
</property>
<property title="SpinMultiplicity" dictRef="me:spinMultiplicity">
<scalar>2.00 </scalar>
</property>
<property title="Vibrational Frequencies" dictRef="me:vibFreqs">
<array units="cm-1">1.87 103.66 128.65 141.60 216.10 220.04 242.13 319.02 689.69 821.49 998.26 1347.91 1518.56 1625.07 2220.76 3121.91 3882.16 3980.71 </array>
</property>
<property title="Rotational Constants" dictRef="me:rotConsts">
<array units="cm-1">0.233 0.100 0.071 </array>
</property>
<property title="Symmetry Number" dictRef="me:symmetryNumber">
<scalar>1 </scalar>
</property>
</propertyList>
<me:deltaEDown units="cm-1" >250.0</me:deltaEDown>
<me:deltaEDownTExponent >0.4</me:deltaEDownTExponent>
</molecule>

<molecule xmlns="http://www.xml-cml.org/schema" id="TS1" spinMultiplicity="2">
<atomArray>
<atom id="a1" elementType="C" x3="-1.408077" y3="0.001881" z3="0.000017"/>
<atom id="a2" elementType="H" x3="-2.171424" y3="0.799563" z3="0.000068"/>
<atom id="a3" elementType="C" x3="0.040107" y3="0.520642" z3="-0.000018"/>
<atom id="a4" elementType="H" x3="0.846935" y3="-0.302663" z3="-0.000054"/>
<atom id="a5" elementType="O" x3="-1.654933" y3="-1.161319" z3="-0.000010"/>

<atom id="a6" elementType="O" x3="0.286440" y3="1.680995" z3="-
0.000002"/>
<atom id="a7" elementType="O" spinMultiplicity="2" x3="2.217169" y3="-
0.956173" z3="0.000009"/>
<atom id="a8" elementType="H" x3="2.742907" y3="-0.140057"
z3="0.000018"/>
</atomArray>
<bondArray>
<bond atomRefs2="a4 a3" order="1"/>
<bond atomRefs2="a3 a6" order="2"/>
<bond atomRefs2="a3 a1" order="1"/>
<bond atomRefs2="a5 a1" order="2"/>
<bond atomRefs2="a7 a8" order="1"/>
<bond atomRefs2="a1 a2" order="1"/>
</bondArray>
<propertyList>
<property title="program">
<scalar>Gaussian 09, Revision D.01</scalar>
</property>
<property title="basis">
<scalar>6-311+G(3df,2pd) (5D, 7F)</scalar>
</property>
<property title="File Format">
<scalar>g03</scalar>
</property>
<property title="Energy" dictRef="me:ZPE">
<scalar units="kJ/mol" >130.2</scalar>
</property>
<property dictRef="me:imaginaryFrequencyScaleFactor" default="true">
<scalar>1</scalar>
</property>
<property title="SpinMultiplicity" dictRef="me:spinMultiplicity">
<scalar>2.00 </scalar>
</property>
<property title="Vibrational Frequencies" dictRef="me:vibFreqs">
<array units="cm-1">56.49 77.45 111.62 193.33 269.70 542.89 667.43 809.45
906.39 1069.84 1267.70 1362.89 1746.38 1901.92 1909.22 2985.71 3796.19 </array>
</property>
<property title="ImaginaryFrequency" dictRef="me:imFreqs">
<scalar units="cm-1">662.79 </scalar>
</property>
<property title="Rotational Constants" dictRef="me:rotConsts">
<array units="cm-1">0.197 0.105 0.068 </array>
</property>
<property title="Symmetry Number" dictRef="me:symmetryNumber">
<scalar>1 </scalar>
</property>

```
    </propertyList>

  </molecule>

  <molecule xmlns="http://www.xml-cml.org/schema" id="C2" spinMultiplicity="2">
    <atomArray>
      <atom id="a1" elementType="C" spinMultiplicity="3" x3="0.202227"
y3="0.619571" z3="-0.001308"/>
      <atom id="a2" elementType="H" x3="-2.543369" y3="-0.875411"
z3="0.811111"/>
      <atom id="a3" elementType="C" x3="0.892783" y3="-0.594844" z3="-
0.023778"/>
      <atom id="a4" elementType="H" x3="0.260186" y3="-1.480395" z3="-
0.074860"/>
      <atom id="a5" elementType="O" x3="2.135271" y3="-0.577513"
z3="0.011170"/>
      <atom id="a6" elementType="O" spinMultiplicity="2" x3="-0.252284"
y3="1.670770" z3="0.015351"/>
      <atom id="a7" elementType="O" x3="-2.079583" y3="-0.771427" z3="-
0.022468"/>
      <atom id="a8" elementType="H" x3="-2.714112" y3="-0.367193" z3="-
0.618167"/>
    </atomArray>
    <bondArray>
      <bond atomRefs2="a8 a7" order="1"/>
      <bond atomRefs2="a4 a3" order="1"/>
      <bond atomRefs2="a3 a1" order="1"/>
      <bond atomRefs2="a3 a5" order="2"/>
      <bond atomRefs2="a7 a2" order="1"/>
      <bond atomRefs2="a1 a6" order="1"/>
    </bondArray>
    <propertyList>
      <property title="program">
        <scalar>Gaussian 09, Revision D.01</scalar>
      </property>
      <property title="basis">
        <scalar>6-311+G(3df,2pd) (5D, 7F)</scalar>
      </property>
      <property title="File Format">
        <scalar>g03</scalar>
      </property>
      <property title="Energy" dictRef="me:ZPE">
        <scalar units="kJ/mol">-18.7 </scalar>
      </property>
      <property title="SpinMultiplicity" dictRef="me:spinMultiplicity">
        <scalar>2.00 </scalar>
      </property>
```

```
      <property title="Vibrational Frequencies" dictRef="me:vibFreqs">
        <array units="cm-1">80.87 103.66 128.65 141.60 216.10 220.04 242.13 319.02
689.69 821.49 998.26 1347.91 1518.56 1625.07 2220.76 3121.91 3882.16 3980.71
</array>
      </property>
      <property title="Rotational Constants" dictRef="me:rotConsts">
        <array units="cm-1">0.233 0.100 0.071 </array>
      </property>
      <property title="Symmetry Number" dictRef="me:symmetryNumber">
        <scalar>1 </scalar>
      </property>
    </propertyList>
    <me:deltaEDown units="cm-1" >250.0</me:deltaEDown>

  </molecule>

  <molecule id="HC(O)CO" description="CHOCO">
    <atomArray>
      <atom id="a1" elementType="C"/>
      <atom id="a2" elementType="H"/>
      <atom id="a3" elementType="O"/>
      <atom id="a4" elementType="C"/>
      <atom id="a5" elementType="O"/>
    </atomArray>
    <bondArray>
      <bond atomRefs2="a1 a2" order="1"/>
      <bond atomRefs2="a1 a3" order="2"/>
      <bond atomRefs2="a1 a4" order="1"/>
      <bond atomRefs2="a4 a5" order="2"/>
    </bondArray>
    <propertyList>
      <property dictRef="me:ZPE">
        <scalar units="kJ/mol">0</scalar>
      </property>
      <property dictRef="me:rotConsts">
        <array units="cm-1">1.995 0.168 0.155</array>
      </property>
      <property dictRef="me:symmetryNumber">
        <scalar>1</scalar>
      </property>
      <property dictRef="me:frequenciesScaleFactor">
        <scalar>0.97</scalar>
      </property>
      <property dictRef="me:vibFreqs">
        <array units="cm-1">200.93 287.30 701.68 795.54 1025.42 1359.35 1530.11
2250.03 3119.43 </array>
      </property>
```

```
      <property dictRef="me:MW">
        <scalar units="amu">57</scalar>
      </property>
      <property dictRef="me:spinMultiplicity">
        <scalar>2</scalar>
      </property>
      <property dictRef="me:epsilon">
        <scalar>216.11</scalar>
      </property>
      <property dictRef="me:sigma">
        <scalar>4.6</scalar>
      </property>
    </propertyList>

    <me:deltaEDown upper="500" lower="50" stepsize="0.1"
>98.3</me:deltaEDown>
    <me:deltaEDownTExponent  >1</me:deltaEDownTExponent>

    <me:DOSCMethod xsi:type="ClassicalRotors"/>
  </molecule>

  <molecule id="H2O">
    <propertyList>
      <property dictRef="me:ZPE">
        <scalar   units="kJ/mol">0</scalar>
      </property>

      <property dictRef="me:rotConsts">
        <array units="cm-1">27.3561325 14.67890037 9.552935159 </array>
      </property>
      <property dictRef="me:vibFreqs">
        <array units="cm-1">1622 3860 3989  </array>
      </property>
      <property dictRef="me:frequenciesScaleFactor">
        <scalar  >1</scalar>
      </property>
      <property dictRef="me:symmetryNumber">
        <scalar>2</scalar>
      </property>
      <property dictRef="me:MW">
        <scalar units="amu">18</scalar>
      </property>
      <property dictRef="me:spinMultiplicity">
        <scalar>1</scalar>
      </property>
    </propertyList>
    <me:DOSCMethod xsi:type="ClassicalRotors"/>
```

</molecule>

<molecule xmlns="http://www.xml-cml.org/schema" id="TS2"
spinMultiplicity="2">
<atomArray>
<atom id="a1" elementType="C" spinMultiplicity="2" x3="0.928961"
y3="0.424489" z3="-0.185109"/>
<atom id="a2" elementType="C" spinMultiplicity="3" x3="-0.974069"
y3="0.333304" z3="0.375054"/>
<atom id="a3" elementType="O" x3="-1.749052" y3="-0.282181" z3="-
0.183675"/>
<atom id="a4" elementType="O" x3="1.631758" y3="-0.472693"
z3="0.075401"/>
<atom id="a5" elementType="H" x3="1.209005" y3="1.492234" z3="-
0.273473"/>
</atomArray>
<bondArray>
<bond atomRefs2="a5 a1" order="1"/>
<bond atomRefs2="a1 a4" order="2"/>
<bond atomRefs2="a3 a2" order="2"/>
<bond id="b1" atomRefs2="a1 a2" order="1"/>
</bondArray>
<propertyList>
<property title="program">
<scalar>Gaussian 09, Revision C.01</scalar>
</property>
<property dictRef="me:imaginaryFrequencyScaleFactor" default="true">
<scalar>1</scalar>
</property>
<property title="basis">
<scalar>6-311+G(3df,2pd) (5D, 7F)</scalar>
</property>
<property title="File Format">
<scalar>g03</scalar>
</property>
<property title="Energy" dictRef="me:ZPE">
<scalar upper="39" lower="35" stepsize="0.1">37.3</scalar>
</property>
<property title="SpinMultiplicity" dictRef="me:spinMultiplicity">
<scalar>2.00 </scalar>
</property>
<property title="Vibrational Frequencies" dictRef="me:vibFreqs">
<array units="cm-1"> 250.60 328.12 634.79 1177.70 1942.05 2107.52 2902.81
</array>
</property>

<property title="ImaginaryFrequency" dictRef="me:imFreqs">

```
<scalar units="cm-1">379.65 </scalar>
</property>
<property title="Rotational Constants" dictRef="me:rotConsts">
<array units="cm-1">1.265 0.143 0.134 </array>
</property>
<property title="Symmetry Number" dictRef="me:symmetryNumber">
<scalar>1 </scalar>
</property>
</propertyList>
<me:DOSCMethod xsi:type="ClassicalRotors"/>
<me:ExtraDOSCMethod xsi:type="me:HinderedRotorQM1D">
<me:bondRef>b1</me:bondRef>
<me:HinderedRotorPotential format="numerical" units="kJ/mol"
expansionSize="7">
<me:PotentialPoint angle="  10  " potential=" 0 "/>
<me:PotentialPoint angle="  20  " potential=" 0.027533619 "/>
<me:PotentialPoint angle="  30  " potential=" 0.385489037 "/>
<me:PotentialPoint angle="  40  " potential=" 1.071463924 "/>
<me:PotentialPoint angle="  50  " potential=" 2.026998897 "/>
<me:PotentialPoint angle="  60  " potential=" 3.079900536 "/>
<me:PotentialPoint angle=" 70 " potential=" 4.023754659 "/>
<me:PotentialPoint angle="  80  " potential=" 4.704349896 "/>
<me:PotentialPoint angle="  90  " potential=" 5.44173995 "/>
<me:PotentialPoint angle="  100  " potential=" 5.05148563 "/>
<me:PotentialPoint angle="  110  " potential=" 4.628635727 "/>
<me:PotentialPoint angle="  120  " potential=" 3.905859207 "/>
<me:PotentialPoint angle="  130  " potential=" 2.935907614 "/>
<me:PotentialPoint angle="  140  " potential=" 1.884127063 "/>
<me:PotentialPoint angle="  150  " potential=" 0.958052827 "/>
<me:PotentialPoint angle="  160  " potential=" 0.317034376 "/>
<me:PotentialPoint angle="  170  " potential=" 0.004851924 "/>
<me:PotentialPoint angle="  180  " potential=" 0.019071632 "/>
<me:PotentialPoint angle="  190  " potential=" 0.307900261 "/>
<me:PotentialPoint angle="  200  " potential=" 0.843410369 "/>
<me:PotentialPoint angle="  210  " potential=" 1.600982641 "/>
<me:PotentialPoint angle="  220  " potential=" 2.510936307 "/>
<me:PotentialPoint angle="  230  " potential=" 3.4892895 "/>
<me:PotentialPoint angle="  240  " potential=" 4.252758645 "/>
<me:PotentialPoint angle="  250  " potential=" 4.364738845 "/>
<me:PotentialPoint angle="  260  " potential=" 4.4 "/>
<me:PotentialPoint angle=" 270 " potential=" 4.42 "/>
<me:PotentialPoint angle="  280  " potential=" 4.4 "/>
<me:PotentialPoint angle="  290  " potential=" 4.364738845 "/>
<me:PotentialPoint angle="  300  " potential=" 4.252758645 "/>
<me:PotentialPoint angle="  310  " potential=" 3.4892895 "/>
<me:PotentialPoint angle="  320  " potential=" 2.510936307 "/>
<me:PotentialPoint angle="  330  " potential=" 1.600982641 "/>
```

```
    &lt;me:PotentialPoint angle="  340  " potential=" 0.843410369 "/&gt;
    &lt;me:PotentialPoint angle="  350  " potential=" 0.307900261 "/&gt;
    &lt;me:PotentialPoint angle="  360  " potential=" 0.019071632 "/&gt;
    &lt;me:PotentialPoint angle="  370  " potential=" 0 "/&gt;
   &lt;/me:HinderedRotorPotential&gt;
  &lt;/me:ExtraDOSCMethod&gt;
&lt;/molecule&gt;

&lt;molecule id="CO" description="Carbon monoxide"&gt;
  &lt;atomArray&gt;
   &lt;atom id="a1" elementType="C"/&gt;
   &lt;atom id="a2" elementType="O"/&gt;
  &lt;/atomArray&gt;
  &lt;bondArray&gt;
   &lt;bond atomRefs2="a1 a2" order="3"/&gt;
  &lt;/bondArray&gt;
  &lt;propertyList&gt;
   &lt;property dictRef="me:ZPE"&gt;
    &lt;scalar units="kJ/mol"&gt;-8.37&lt;/scalar&gt;
   &lt;/property&gt;
   &lt;property dictRef="me:rotConsts"&gt;
    &lt;array units="cm-1"/&gt;
   &lt;/property&gt;
   &lt;property dictRef="me:symmetryNumber"&gt;
    &lt;scalar&gt;1&lt;/scalar&gt;
   &lt;/property&gt;
   &lt;property dictRef="me:frequenciesScaleFactor"&gt;
    &lt;scalar&gt;0.9854&lt;/scalar&gt;
   &lt;/property&gt;
   &lt;property dictRef="me:vibFreqs"&gt;
    &lt;array units="cm-1"/&gt;
   &lt;/property&gt;
   &lt;property dictRef="me:MW"&gt;
    &lt;scalar units="amu"&gt;28&lt;/scalar&gt;
   &lt;/property&gt;
   &lt;property dictRef="me:spinMultiplicity"&gt;
    &lt;scalar&gt;2&lt;/scalar&gt;
   &lt;/property&gt;
  &lt;/propertyList&gt;
&lt;/molecule&gt;

&lt;molecule id="HCO" description="Carbon monoxide"&gt;
  &lt;atomArray&gt;
   &lt;atom id="a1" elementType="C"/&gt;
   &lt;atom id="a2" elementType="O"/&gt;
   &lt;atom id="a2" elementType="H"/&gt;
  &lt;/atomArray&gt;
```

```
    <bondArray>
      <bond atomRefs2="a1 a2" order="3"/>
      <bond atomRefs2="a1 a3" order="1"/>
    </bondArray>
    <propertyList>
      <property dictRef="me:ZPE">
        <scalar units="kJ/mol">0</scalar>
      </property>
      <property dictRef="me:rotConsts">
        <array units="cm-1"/>
      </property>
      <property dictRef="me:symmetryNumber">
        <scalar>1</scalar>
      </property>
      <property dictRef="me:frequenciesScaleFactor">
        <scalar>0.9854</scalar>
      </property>
      <property dictRef="me:vibFreqs">
        <array units="cm-1"/>
      </property>
      <property dictRef="me:MW">
        <scalar units="amu">29</scalar>
      </property>
      <property dictRef="me:spinMultiplicity">
        <scalar>2</scalar>
      </property>
    </propertyList>
    <me:DOSCMethod xsi:type="ClassicalRotors"/>
  </molecule>

  <molecule xmlns="http://www.xml-cml.org/schema" id="TS3" spinMultiplicity="2">
    <atomArray>
      <atom id="a1" elementType="C" x3="-0.438261" y3="-1.150675" z3="0.369974"/>
      <atom id="a2" elementType="C" spinMultiplicity="2" x3="-0.972277" y3="0.171806" z3="0.074828"/>
      <atom id="a3" elementType="O" x3="-1.802174" y3="0.899718" z3="-0.234216"/>
      <atom id="a4" elementType="O" x3="0.365111" y3="-1.661436" z3="-0.372163"/>
      <atom id="a5" elementType="O" x3="0.787364" y3="1.161442" z3="0.171340"/>
      <atom id="a6" elementType="O" x3="1.791615" y3="0.521735" z3="-0.071751"/>
      <atom id="a7" elementType="H" x3="-0.672108" y3="-1.498463" z3="1.385507"/>
```

</atomArray>
<bondArray>
<bond atomRefs2="a4 a1" order="2"/>
<bond atomRefs2="a3 a2" order="2"/>
<bond atomRefs2="a6 a5" order="2"/>
<bond id="b1" atomRefs2="a2 a5" order="2"/>
<bond id="b2" atomRefs2="a2 a1" order="1"/>
<bond atomRefs2="a1 a7" order="1"/>
</bondArray>
<propertyList>
<property dictRef="me:imaginaryFrequencyScaleFactor" default="true">
<scalar>1</scalar>
</property>
<property title="program">
<scalar>Gaussian 09, Revision D.01</scalar>
</property>
<property title="basis">
<scalar>6-311+G(3df,2pd) (5D, 7F)</scalar>
</property>
<property title="File Format">
<scalar>g03</scalar>
</property>
<property title="Energy" dictRef="me:ZPE">
<scalar units="kJ/mol" >0.5 </scalar>
</property>
<property title="SpinMultiplicity" dictRef="me:spinMultiplicity">
<scalar>2.00 </scalar>
</property>
<property title="Vibrational Frequencies" dictRef="me:vibFreqs">
<array units="cm-1">104.83 147.71 181.99 253.28 287.83 389.17 655.69 817.77 926.11 1368.01 1460.95 1725.86 2143.84 3023.74 </array>
</property>
<property title="ImaginaryFrequency" dictRef="me:imFreqs">
<scalar units="cm-1">526.63 </scalar>
</property>
<property title="Hessian" dictRef="me:hessian">
<matrix rows="21" matrixType="squareSymmetricLT" units="Hartree/Bohr2">0.35017584 0.30048587 0.70154378 0.10981205
0.01688383 0.44870476 -0.08436764 -0.03238940 -0.06678640 0.35903268 -
0.01215968 -0.07457154 0.02561638 -0.02291337 0.13302487 0.01145414
0.08388188 -0.12046697 0.61198956 -0.04328586 0.93803934 0.01367662
0.02451724 0.00819589 -0.37901675 0.03376026 -0.51342484 0.40048699
0.00618712 0.02115630 -0.01050192 0.03727412 -0.04614606 0.05324158 -
0.03909563 0.02174557 -0.01931278 0.00068839 -0.02679191 -0.50612940
0.04950289 -0.72835720 0.52006756 -0.05315946 0.75895359 -0.22740441 -
0.27238442 -0.10435817 0.03330442 -0.01516358 -0.06701275 -0.01519587

0.00140174 -0.00128345 0.22905320 -0.25234214 -0.43965609 -0.13726460
0.02194574 -0.00631805 -0.08768594 -0.01744388 0.00141831 0.00025376
0.30425517 0.47923420 -0.13302231 -0.19813506 -0.15942842 -0.01340783 -
0.04247092 -0.06328736 -0.00304502 0.00832759 -0.00089023 0.16419837
0.22993565 0.19410703 0.04940101 0.02753950 0.07912591 0.09423228
0.04089305 -0.11143491 0.00058457 -0.01356705 0.03998863 -0.05118816 -
0.04845694 -0.03451282 0.19918755 0.02387817 0.01421897 0.02119002
0.01788789 -0.00361466 -0.04623985 0.00604486 0.00042877 0.01740948 -
0.01647406 -0.01399420 -0.00958294 0.13987391 0.10300776 0.03401521
0.01242656 0.02442145 0.03232211 -0.00412943 -0.08466150 -0.01151746
0.00212079 0.02533830 -0.02021703 -0.02067460 -0.01308579 0.24433190
0.17524670 0.33358893 -0.04520306 -0.01384715 -0.03908870 -0.03341391 -
0.02368963 0.06130321 -0.01935433 0.00810112 -0.02948267 0.02855596
0.02241497 0.01496222 -0.29317234 -0.17061344 -0.27710908 0.36057296 -
0.02274721 -0.00655855 -0.02115883 -0.01908940 -0.00868666 0.04037900 -
0.00688158 0.00382696 -0.01663698 0.01779945 0.00990557 0.00826817 -
0.14919655 -0.10053951 -0.16714139 0.18004259 0.10268015 -0.02848907 -
0.01707146 -0.04129750 -0.04413141 -0.01234021 0.07694352 -0.00422737
0.00422033 -0.02721328 0.03015575 0.02797057 0.01757987 -0.22577639 -
0.15810292 -0.28665586 0.27257748 0.15700569 0.26303782 -0.05627836 -
0.03392164 0.01309942 0.01022892 -0.00072703 0.00712561 -0.00118122 -
0.00030142 -0.00384790 0.00287486 -0.03037292 0.00482738 0.00095509 -
0.00059733 -0.00182565 0.00201472 0.00007270 -0.00010899 0.04138600 -
0.04330212 -0.21613286 0.10523511 -0.00271558 0.00631211 -0.00029081 -
0.00090127 -0.00242985 0.00194193 -0.01943430 -0.03058973 0.00365751
0.00291408 0.00049288 0.00215137 -0.00240845 -0.00062795 -0.00168200
0.06584764 0.24297539 0.02554276 0.10132586 -0.12514140 -0.01385662
0.02710715 -0.01820983 0.00395123 -0.00424891 -0.00103927 -0.00148272 -
0.01253484 0.02500490 0.00827768 0.00007951 0.00105447 -0.00316246 -
0.00071566 -0.00239456 -0.01926986 -0.11101311 0.12072570 </matrix>
</property>
<property title="Rotational Constants" dictRef="me:rotConsts">
<array units="cm-1">0.155 0.123 0.073 </array>
</property>
<property title="Symmetry Number" dictRef="me:symmetryNumber">
<scalar>1 </scalar>
</property>
</propertyList>
<me:ExtraDOSCMethod xsi:type="me:HinderedRotorQM1D">
<me:bondRef> b1 </me:bondRef>
<me:HinderedRotorPotential format="numerical" expansionSize="15" units="kJ/mol" useSineTerms="yes">
<me:PotentialPoint angle= "0" potential = "0.0" />
<me:PotentialPoint angle= "10" potential = "2.15536484244" />
<me:PotentialPoint angle= "20" potential = "4.87058605393" />
<me:PotentialPoint angle= "30" potential = "7.51716356793" />
<me:PotentialPoint angle= "40" potential = "9.31663400901" />

<me:PotentialPoint angle= "50" potential = "10.2685799224" />
<me:PotentialPoint angle= "60" potential = "10.0752748594" />
<me:PotentialPoint angle= "70" potential = "8.51835861039" />
<me:PotentialPoint angle= "80" potential = "6.16698706547" />
<me:PotentialPoint angle= "90" potential = "3.44434881642" />
<me:PotentialPoint angle= "100" potential = "0.683223362911" />
<me:PotentialPoint angle= "110" potential = "-1.87522661806" />
<me:PotentialPoint angle= "120" potential = "-3.56359640108" />
<me:PotentialPoint angle= "130" potential = "-4.33156040203" />
<me:PotentialPoint angle= "140" potential = "-4.27483122351" />
<me:PotentialPoint angle= "150" potential = "-3.16316564304" />
<me:PotentialPoint angle= "160" potential = "-1.2386347606" />
<me:PotentialPoint angle= "170" potential = "0.987542442527" />
<me:PotentialPoint angle= "180" potential = "3.51727732996" />
<me:PotentialPoint angle= "190" potential = "5.46932607792" />
<me:PotentialPoint angle= "200" potential = "6.41310406097" />
<me:PotentialPoint angle= "210" potential = "6.14206844493" />
<me:PotentialPoint angle= "220" potential = "4.09987052898" />
<me:PotentialPoint angle= "230" potential = "1.42632387898" />
<me:PotentialPoint angle= "240" potential = "0.181464057904" />
<me:PotentialPoint angle= "250" potential = "0.801092560019" />
<me:PotentialPoint angle= "260" potential = "2.88807100395" />
<me:PotentialPoint angle= "270" potential = "7.44264400151" />
<me:PotentialPoint angle= "280" potential = "9.34336422449" />
<me:PotentialPoint angle= "290" potential = "4.35347545039" />
<me:PotentialPoint angle= "300" potential = "1.93414021246" />
<me:PotentialPoint angle= "310" potential = "-0.0193998195474" />
<me:PotentialPoint angle= "320" potential = "-1.28049310708" />
<me:PotentialPoint angle= "330" potential = "-2.14674270059" />
<me:PotentialPoint angle= "340" potential = "-2.14410407309" />
<me:PotentialPoint angle= "350" potential = "-1.42511614907" />
<me:PotentialPoint angle= "360" potential = "-0.000147028086786" />
</me:HinderedRotorPotential>
</me:ExtraDOSCMethod>
<me:ExtraDOSCMethod xsi:type="HinderedRotorQM1D">
<me:bondRef> b2 </me:bondRef>
<me:HinderedRotorPotential format="numerical" expansionSize="15" units="kJ/mol" useSineTerms="yes">
<me:PotentialPoint angle= "0" potential = "0.0" />
<me:PotentialPoint angle= "10" potential = "-0.538004332606" />
<me:PotentialPoint angle= "20" potential = "0.547135821447" />
<me:PotentialPoint angle= "30" potential = "2.97196097988" />
<me:PotentialPoint angle= "40" potential = "6.47679606544" />
<me:PotentialPoint angle= "50" potential = "10.9037172529" />
<me:PotentialPoint angle= "60" potential = "15.7018578854" />
<me:PotentialPoint angle= "70" potential = "20.6029233689" />
<me:PotentialPoint angle= "80" potential = "24.5671185599" />

```
        <me:PotentialPoint angle= "90" potential = "27.319214919" />
        <me:PotentialPoint angle= "100" potential = "5.02751481442" />
        <me:PotentialPoint angle= "110" potential = "1.96889920695" />
        <me:PotentialPoint angle= "120" potential = "-0.0670710230462" />
        <me:PotentialPoint angle= "130" potential = "-0.505702806091" />
        <me:PotentialPoint angle= "140" potential = "0.591154954414" />
        <me:PotentialPoint angle= "150" potential = "3.03971725849" />
        <me:PotentialPoint angle= "160" potential = "5.9170289635" />
        <me:PotentialPoint angle= "170" potential = "8.34330864891" />
        <me:PotentialPoint angle= "180" potential = "9.9748941179" />
        <me:PotentialPoint angle= "190" potential = "11.3097902105" />
        <me:PotentialPoint angle= "200" potential = "12.6832365195" />
        <me:PotentialPoint angle= "210" potential = "3.24202516089" />
        <me:PotentialPoint angle= "220" potential = "2.70268182342" />
        <me:PotentialPoint angle= "360" potential = "0.0" />
      </me:HinderedRotorPotential>
    </me:ExtraDOSCMethod>

  </molecule>

  <molecule xmlns="http://www.xml-cml.org/schema" id="R02"
spinMultiplicity="2">
    <atomArray>
      <atom id="a1" elementType="C" x3="-1.464804" y3="0.279042" z3="-
0.000006"/>
      <atom id="a2" elementType="C" x3="0.054604" y3="0.462790" z3="-
0.000001"/>
      <atom id="a3" elementType="O" x3="0.595206" y3="1.508921"
z3="0.000004"/>
      <atom id="a4" elementType="O" x3="-1.991164" y3="-0.786044"
z3="0.000007"/>
      <atom id="a5" elementType="O" x3="0.702112" y3="-0.773840" z3="-
0.000006"/>
      <atom id="a6" elementType="O" spinMultiplicity="2" x3="2.001636" y3="-
0.660476" z3="0.000002"/>
      <atom id="a7" elementType="H" x3="-2.001126" y3="1.240519" z3="-
0.000021"/>
    </atomArray>
    <bondArray>
      <bond atomRefs2="a7 a1" order="1"/>
      <bond id="b1" atomRefs2="a1 a2" order="1"/>
      <bond atomRefs2="a1 a4" order="2"/>
      <bond id="b2" atomRefs2="a5 a2" order="1"/>
      <bond atomRefs2="a5 a6" order="1"/>
      <bond atomRefs2="a2 a3" order="2"/>
    </bondArray>
    <propertyList>
```

<property title="program">
<scalar>Gaussian 09, Revision C.01</scalar>
</property>
<property title="basis">
<scalar>6-311+G(3df,2pd) (5D, 7F)</scalar>
</property>
<property title="File Format">
<scalar>g03</scalar>
</property>
<property title="Energy" dictRef="me:ZPE">
<scalar units="kJ/mol" >-112.7 </scalar>
</property>
<property title="SpinMultiplicity" dictRef="me:spinMultiplicity">
<scalar>2.00 </scalar>
</property>

<property title="Vibrational Frequencies" dictRef="me:vibFreqs">
<array units="cm-1">213.01 370.52 522.34 541.87 704.04 866.04 1020.15
1201.93 1226.01 1382.95 1894.89 1942.01 3022.46 </array>
</property>
<property title="Rotational Constants" dictRef="me:rotConsts">
<array units="cm-1">0.248 0.099 0.071 </array>
</property>
<property title="Symmetry Number" dictRef="me:symmetryNumber">
<scalar>1 </scalar>
</property>
<property dictRef="me:epsilon">
<scalar>216.11</scalar>
</property>
<property dictRef="me:sigma">
<scalar>4.6</scalar>
</property>
</propertyList>
<me:deltaEDown units="cm-1" >250.0</me:deltaEDown>
<me:deltaEDownTExponent >0.0</me:deltaEDownTExponent>
<me:ExtraDOSCMethod xsi:type="me:HinderedRotorQM1D">
<me:bondRef>b1</me:bondRef>
<me:HinderedRotorPotential format="numerical" units="kJ/mol"
expansionSize="7">
<me:PotentialPoint angle=" 0 " potential=" 0 "/>
<me:PotentialPoint angle=" 36 " potential="
3.520259898 "/>
<me:PotentialPoint angle=" 72 " potential="
12.40227126 "/>
<me:PotentialPoint angle=" 108 " potential="
15.35504771 "/>

<me:PotentialPoint angle=" 144 " potential="
8.942823195 "/>
<me:PotentialPoint angle=" 180 " potential="
5.154796429 "/>
<me:PotentialPoint angle=" 216 " potential="
8.942828446 "/>
<me:PotentialPoint angle=" 252 " potential="
15.35489281 "/>
<me:PotentialPoint angle=" 288 " potential="
12.40225551 "/>
<me:PotentialPoint angle=" 324 " potential="
3.520280902 "/>
<me:PotentialPoint angle=" 360 " potential=" -
0.127748954 "/>
</me:HinderedRotorPotential>
</me:ExtraDOSCMethod>
<me:ExtraDOSCMethod xsi:type="HinderedRotorQM1D">
<me:bondRef>b2</me:bondRef>
<me:HinderedRotorPotential format="numerical" units="kJ/mol"
expansionSize="7">
<me:PotentialPoint angle=" 0 " potential=" 0 "/>
<me:PotentialPoint angle=" 36 " potential="
9.401965384 "/>
<me:PotentialPoint angle=" 72 " potential="
24.38858456 "/>
<me:PotentialPoint angle=" 108 " potential="
17.53182876 "/>
<me:PotentialPoint angle=" 144 " potential="
7.588485275 "/>
<me:PotentialPoint angle=" 180 " potential="
11.81304343 "/>
<me:PotentialPoint angle=" 216 " potential="
7.588569291 "/>
<me:PotentialPoint angle=" 252 " potential="
17.5318839 "/>
<me:PotentialPoint angle=" 288 " potential="
24.38853468 "/>
<me:PotentialPoint angle=" 324 " potential="
9.401823607 "/>
<me:PotentialPoint angle=" 360 " potential=" -
0.127882854 "/>
</me:HinderedRotorPotential>
</me:ExtraDOSCMethod>
</molecule>

<molecule xmlns="http://www.xml-cml.org/schema" id="TS4"
spinMultiplicity="2">

<atomArray>
<atom id="a1" elementType="C" x3="-0.811217" y3="-0.645788" z3="0.000004"/>
<atom id="a2" elementType="C" x3="0.058189" y3="0.670192" z3="-0.000002"/>
<atom id="a3" elementType="O" x3="-0.315226" y3="1.782606" z3="-0.000007"/>
<atom id="a4" elementType="O" x3="-1.967052" y3="-0.757635" z3="0.000004"/>
<atom id="a5" elementType="O" x3="1.338490" y3="0.257152" z3="0.000000"/>
<atom id="a6" elementType="O" spinMultiplicity="2" x3="1.493921" y3="-1.120299" z3="0.000001"/>
<atom id="a7" elementType="H" x3="0.117109" y3="-1.441012" z3="0.000008"/>
</atomArray>
<bondArray>
<bond atomRefs2="a3 a2" order="2"/>
<bond id="b2" atomRefs2="a2 a5" order="1"/>
<bond id="b1" atomRefs2="a2 a1" order="1"/>
<bond atomRefs2="a5 a6" order="1"/>
<bond atomRefs2="a1 a4" order="2"/>
<bond atomRefs2="a1 a7" order="1"/>
</bondArray>
<propertyList>
<property title="program">
<scalar>Gaussian 09, Revision C.01</scalar>
</property>
<property dictRef="me:imaginaryFrequencyScaleFactor" default="true">
<scalar>1</scalar>
</property>
<property title="basis">
<scalar>6-311+G(3df,2pd) (5D, 7F)</scalar>
</property>
<property title="File Format">
<scalar>g03</scalar>
</property>
<property title="Energy" dictRef="me:ZPE">
<scalar units="kJ/mol" >-43.5 </scalar>
</property>
<property title="SpinMultiplicity" dictRef="me:spinMultiplicity">
<scalar>2.00 </scalar>
</property>
<property title="Vibrational Frequencies" dictRef="me:vibFreqs">
<array units="cm-1">47.29 164.51 302.98 492.79 520.95 630.69 655.57 797.92 1018.27 1039.46 1129.77 1911.20 2011.36 2061.71 </array>
</property>

<property title="ImaginaryFrequency" dictRef="me:imFreqs">
<scalar units="cm-1">1137.92 </scalar>
</property>
<property title="Hessian" dictRef="me:hessian">
<matrix rows="21" matrixType="squareSymmetricLT" units="Hartree/Bohr2">1.14189504 0.06212667 0.27812980 0.00000059 -0.00000060 0.12360617 0.00817989 -0.04577419 0.00000024 0.45135977 0.02531433 -0.05554055 -0.00000007 -0.24047179 1.04217342 -0.00000011 -0.00000005 -0.07649033 0.00000103 -0.00000350 0.13552168 -0.00455386 -0.00099474 -0.00000001 -0.14453943 0.23216788 -0.00000090 0.18314491 0.00717341 -0.05209269 0.00000029 0.22553666 -0.81935348 0.00000296 -0.29631103 0.89911460 0.00000001 0.00000026 0.01962050 -0.00000089 0.00000295 -0.05617689 0.00000118 -0.00000344 0.02630292 -0.99945284 -0.09197909 -0.00000046 -0.05669385 -0.01594463 0.00000006 0.00940517 -0.00868015 0.00000001 1.07236773 -0.08667824 -0.07936573 0.00000007 -0.02361941 -0.00463410 0.00000006 0.00014492 0.00594175 -0.00000002 0.11641716 0.06510920 -0.00000048 0.00000012 -0.04059721 0.00000010 0.00000007 0.01422369 0.00000000 -0.00000004 -0.00215531 0.00000039 -0.00000014 0.01582953 -0.09633394 0.01460481 -0.00000015 -0.20544594 -0.03380926 -0.00000004 -0.04818318 0.06974419 -0.00000026 0.01427924 -0.01280127 0.00000009 0.36995420 -0.00053836 -0.03769355 0.00000032 0.02286796 -0.08036002 -0.00000011 0.07126420 -0.02865721 0.00000025 -0.01042566 0.00748075 -0.00000004 -0.06434315 0.41642208 -0.00000002 0.00000023 0.01389452 -0.00000034 0.00000019 -0.02842699 -0.00000026 0.00000018 0.01014067 0.00000006 -0.00000003 -0.00034410 0.00000026 -0.00000043 0.00909989 -0.08321962 0.04044682 0.00000009 -0.00433312 0.03397867 -0.00000011 -0.00485035 -0.00055875 0.00000004 -0.00384165 0.00404508 -0.00000013 -0.06245633 0.01817157 0.00000012 0.11288274 -0.04913835 0.02757545 -0.00000018 0.05062049 -0.04589286 0.00000034 -0.00361268 -0.00118820 0.00000000 0.01179862 -0.00478311 0.00000003 0.03920842 -0.27172697 -0.00000020 -0.09686259 0.31684848 0.00000055 -0.00000016 0.01241788 -0.00000015 0.00000003 -0.01674950 -0.00000001 0.00000005 0.00691678 -0.00000004 -0.00000004 -0.00314861 0.00000023 -0.00000002 0.00277917 -0.00000014 0.00000015 0.00259612 0.03348534 0.02156971 -0.00000030 -0.04852731 -0.00123520 0.00000007 0.00957675 0.00309566 -0.00000009 -0.03606379 0.00249176 0.00000004 0.02818596 -0.03699655 0.00000018 0.04581833 0.04798609 -0.00000043 -0.03247527 0.04174054 -0.08101273 0.00000016 0.01084028 -0.03639241 0.00000032 -0.00265855 -0.00376476 0.00000000 -0.00118626 0.01025124 0.00000001 -0.01260374 -0.00546507 0.00000006 0.00077920 -0.02083279 0.00000000 -0.03691147 0.13721652 -0.00000055 0.00000020 -0.05245153 0.00000001 0.00000033 0.02809833 0.00000000 0.00000000 -0.00464866 -0.00000002 0.00000010 0.01619201 -0.00000012 0.00000005 -0.00714316 0.00000015 -0.00000014 -0.00481182 0.00000053 -0.00000055 0.02476484 </matrix>
</property>
<property title="Rotational Constants" dictRef="me:rotConsts">
<array units="cm-1">0.180 0.124 0.073 </array>

</property>
<property title="Symmetry Number" dictRef="me:symmetryNumber">
<scalar>1 </scalar>
</property>
</propertyList>
<me:ExtraDOSCMethod xsi:type="me:HinderedRotorQM1D">
<me:bondRef>b1</me:bondRef>
<me:HinderedRotorPotential format="numerical" units="kJ/mol"
expansionSize="7">
<me:PotentialPoint angle=" 0 " potential=" 0 "/>
<me:PotentialPoint angle=" 36 " potential="
1.944781364 "/>
<me:PotentialPoint angle=" 72 " potential="
7.963327911 "/>
<me:PotentialPoint angle=" 108 " potential="
8.350326611 "/>
<me:PotentialPoint angle=" 144 " potential=" -
0.512455592 "/>
<me:PotentialPoint angle=" 180 " potential=" -
7.192147672 "/>
<me:PotentialPoint angle=" 216 " potential=" -
1.323370147 "/>
<me:PotentialPoint angle=" 252 " potential="
10.2255086 "/>
<me:PotentialPoint angle=" 288 " potential="
11.30181657 "/>
<me:PotentialPoint angle=" 324 " potential=" 4.4428711
"/>
<me:PotentialPoint angle=" 360 " potential=" 0 "/>
</me:HinderedRotorPotential>
</me:ExtraDOSCMethod>

</molecule>

<molecule xmlns="http://www.xml-cml.org/schema" id="QOOH"
spinMultiplicity="2">
<atomArray>
<atom id="a1" elementType="C" spinMultiplicity="2" x3="-0.982945" y3="-
0.394768" z3="0.388914"/>
<atom id="a2" elementType="C" x3="0.021764" y3="0.674650"
z3="0.014621"/>
<atom id="a3" elementType="O" x3="-0.243823" y3="1.827463" z3="-
0.034013"/>
<atom id="a4" elementType="O" x3="-1.849169" y3="-0.909823" z3="-
0.186591"/>
<atom id="a5" elementType="O" x3="1.300468" y3="0.230148" z3="-
0.053875"/>

```
<atom id="a6" elementType="O" x3="1.295292" y3="-1.180761" z3="-
0.116450"/>
<atom id="a7" elementType="H" x3="1.744944" y3="-1.415513"
z3="0.706225"/>
</atomArray>
<bondArray>
<bond atomRefs2="a4 a1" order="2"/>
<bond id="b3" atomRefs2="a6 a5" order="1"/>
<bond atomRefs2="a6 a7" order="1"/>
<bond id="b2" atomRefs2="a5 a2" order="1"/>
<bond atomRefs2="a3 a2" order="2"/>
<bond id="b1" atomRefs2="a2 a1" order="1"/>
</bondArray>
<propertyList>
<property title="program">
<scalar>Gaussian 09, Revision C.01</scalar>
</property>
<property title="basis">
<scalar>6-311+G(3df,2pd) (5D, 7F)</scalar>
</property>
<property title="File Format">
<scalar>g03</scalar>
</property>
<property title="Energy" dictRef="me:ZPE">
<scalar units="kJ/mol" >-136.6 </scalar>
</property>
<property title="SpinMultiplicity" dictRef="me:spinMultiplicity">
<scalar>2.00 </scalar>
</property>
<property title="Vibrational Frequencies" dictRef="me:vibFreqs">
<array units="cm-1">141.76 163.60 252.66 292.71 347.29 479.02 585.88
732.44 736.32 1068.44 1200.29 1444.67 1887.07 2055.92 3826.18 </array>
</property>
<property title="Hessian" dictRef="me:hessian">
<matrix rows="21"  matrixType="squareSymmetricLT"
units="Hartree/Bohr2">0.71477422 0.38037405 0.28692223 0.38319761
0.21157481 0.31613771 -0.08828345 -0.04755376 -0.00909478 0.49765943 -
0.03211060 -0.04325565 -0.03240235 -0.09642696 1.01432436 -0.01563119 -
0.00498393 -0.08340126 -0.00385688 -0.02767528 0.18386425 0.01197058 -
0.00728408 -0.00375813 -0.11170379 0.10609246 0.00137624 0.11758921 -
0.00446444 -0.05181511 0.03767325 0.12283447 -0.81764683 0.03355808 -
0.17162176 0.91543259 0.00809288 0.01713171 0.02557229 -0.00472332
0.03254097 -0.07262515 0.00041924 -0.04735168 0.03556345 -0.60241032 -
0.33250944 -0.38574293 -0.01915818 -0.01116955 -0.00175776 -0.00151131 -
0.01250346 -0.00001394 0.63550175 -0.32548502 -0.19563865 -0.21549780 -
0.02553022 -0.01287373 -0.00376513 -0.00131128 -0.00194828 -0.00152916
0.35701574 0.21282484 -0.39803239 -0.22449624 -0.27669931 0.01243579
```

0.02381594 0.02437958 0.00201645 -0.01961157 -0.00766141 0.39103164
0.21883936 0.26560617 -0.03765033 0.00154334 0.01726604 -0.27758925 -
0.01937887 0.01908830 -0.00923930 0.06229568 -0.00342317 -0.00940334 -
0.00474223 -0.00850532 0.38498921 -0.01403008 -0.00074730 -0.00316869
0.05080008 -0.08783267 0.00235502 0.06295451 -0.04581634 -0.00011567 -
0.00265942 -0.00168332 0.00234200 -0.06466202 0.45498864 0.02333441
0.00531993 0.02232724 0.00010569 0.00490488 -0.04464271 0.00106070 -
0.00570526 0.01233933 -0.00406500 0.00006385 -0.00754167 -0.01024797 -
0.01321521 0.04639988 0.00182062 0.00677783 -0.00063978 0.00336820
0.04704725 0.00294991 -0.00794408 0.00592139 -0.00078615 -0.00295519 -
0.00086339 0.00045931 -0.05508707 -0.02255119 -0.01578542 0.17501099 -
0.00548163 0.00455921 0.00152023 -0.00180467 -0.04675462 -0.00412595
0.00929092 0.00239198 0.00116637 0.00245013 -0.00045098 -0.00041589 -
0.00053613 -0.28626852 -0.02875215 -0.06583510 0.38740684 0.00053341 -
0.00541909 -0.00366126 0.01002070 0.00109096 -0.00888994 -0.00349220
0.00349714 0.00720562 -0.00048382 0.00173549 0.00155788 -0.02364984
0.02147599 -0.03785928 0.22556089 -0.07995804 0.43643609 -0.00022132 -
0.00134793 -0.00122803 -0.00429296 0.00594627 -0.00216863 0.00083869 -
0.00246188 0.00043446 -0.00006341 0.00091640 0.00059453 0.00398008 -
0.00985187 0.00559758 -0.11421346 0.06191648 -0.20848912 0.11397237
0.00119772 -0.00002473 0.00030054 -0.00231894 -0.00596086 0.00463720
0.00187922 -0.00059801 -0.00184254 -0.00062399 -0.00022989 -0.00047359
0.02548023 -0.03264049 0.03738396 0.02950321 -0.06088391 0.05757755 -
0.05511747 0.10033790 -0.00149474 0.00087281 -0.00027540 -0.00488719 -
0.00227512 0.00131522 0.00237771 -0.00205995 -0.00039414 0.00103180
0.00015339 0.00035875 0.00947196 -0.00967344 0.00897721 -0.21175876
0.11056544 -0.39478911 0.20525922 -0.09758313 0.38480747 </matrix>
</property>
<property title="Rotational Constants" dictRef="me:rotConsts">
<array units="cm-1">0.165 0.132 0.075 </array>
</property>
<property title="Symmetry Number" dictRef="me:symmetryNumber">
<scalar>1 </scalar>
</property>
</propertyList>
<me:deltaEDown units="cm-1" >250.0</me:deltaEDown>
<me:deltaEDownTExponent >0.0</me:deltaEDownTExponent>
<me:ExtraDOSCMethod xsi:type="me:HinderedRotorQM1D">
<me:bondRef>b3</me:bondRef>
<me:HinderedRotorPotential format="numerical" units="kJ/mol"
expansionSize="7">
<me:PotentialPoint angle=" 0 " potential=" 0 "/>
<me:PotentialPoint angle=" 36 " potential="
16.03976499 "/>
<me:PotentialPoint angle=" 72 " potential="
41.11543502 "/>

```
<me:PotentialPoint angle=" 108 " potential="
42.58587518 "/>
<me:PotentialPoint angle=" 144 " potential="
14.87418739 "/>
<me:PotentialPoint angle=" 180 " potential=" 0 "/>
<me:PotentialPoint angle=" 216 " potential="
13.49258628 "/>
<me:PotentialPoint angle=" 252 " potential="
28.01572069 "/>
<me:PotentialPoint angle=" 288 " potential="
21.23291472 "/>
<me:PotentialPoint angle=" 324 " potential="
2.41642881 "/>
<me:PotentialPoint angle=" 360 " potential=" 0 "/>

</me:HinderedRotorPotential>
</me:ExtraDOSCMethod>
<me:ExtraDOSCMethod xsi:type="me:HinderedRotorQM1D">
<me:bondRef>b1</me:bondRef>
<me:HinderedRotorPotential format="numerical" units="kJ/mol"
expansionSize="7">
<me:PotentialPoint angle=" 0 " potential=" 0 "/>
<me:PotentialPoint angle=" 36 " potential="
8.378083397 "/>
<me:PotentialPoint angle=" 72 " potential="
25.09082179 "/>
<me:PotentialPoint angle=" 108 " potential="
33.49829766 "/>
<me:PotentialPoint angle=" 144 " potential="
23.5392038 "/>
<me:PotentialPoint angle=" 180 " potential="
9.832266456 "/>
<me:PotentialPoint angle=" 216 " potential="
1.379112138 "/>
<me:PotentialPoint angle=" 252 " potential="
8.320850122 "/>
<me:PotentialPoint angle=" 288 " potential="
25.66219098 "/>
<me:PotentialPoint angle=" 324 " potential="
8.710526832 "/>
<me:PotentialPoint angle=" 360 " potential=" 0 "/>

</me:HinderedRotorPotential>
</me:ExtraDOSCMethod>
<me:ExtraDOSCMethod xsi:type="me:HinderedRotorQM1D">
<me:bondRef>b2</me:bondRef>
```

<me:HinderedRotorPotential format="numerical" units="kJ/mol" expansionSize="7">
<me:PotentialPoint angle=" 0 " potential=" 0 "/>
<me:PotentialPoint angle=" 36 " potential=" 5.658574744 "/>
<me:PotentialPoint angle=" 72 " potential=" 22.00540771 "/>
<me:PotentialPoint angle=" 108 " potential=" 36.77041105 "/>
<me:PotentialPoint angle=" 144 " potential=" 29.78816686 "/>
<me:PotentialPoint angle=" 180 " potential=" 12.41902195 "/>
<me:PotentialPoint angle=" 216 " potential=" 0.894400205 "/>
<me:PotentialPoint angle=" 252 " potential=" 1.869865347 "/>
<me:PotentialPoint angle=" 288 " potential=" 7.341740786 "/>
<me:PotentialPoint angle=" 324 " potential=" 6.449023527 "/>
<me:PotentialPoint angle=" 360 " potential=" 0 "/>

</me:HinderedRotorPotential>
</me:ExtraDOSCMethod>
</molecule>

<molecule xmlns="http://www.xml-cml.org/schema" id="TS5" spinMultiplicity="2">
<atomArray>
<atom id="a1" elementType="C" spinMultiplicity="3" x3="-1.343056" y3="-0.333358" z3="0.453361"/>
<atom id="a2" elementType="C" spinMultiplicity="2" x3="0.348591" y3="0.687451" z3="-0.077525"/>
<atom id="a3" elementType="O" x3="0.379967" y3="1.856247" z3="-0.024574"/>
<atom id="a4" elementType="O" x3="-2.251734" y3="-0.327544" z3="-0.221207"/>
<atom id="a5" elementType="O" x3="1.413899" y3="-0.113168" z3="0.080313"/>
<atom id="a6" elementType="O" x3="1.070904" y3="-1.450912" z3="-0.201730"/>
<atom id="a7" elementType="H" x3="1.062489" y3="-1.841538" z3="0.682571"/>
</atomArray>
<bondArray>
<bond atomRefs2="a4 a1" order="2"/>

<bond id="b2" atomRefs2="a6 a5" order="1"/>
<bond atomRefs2="a6 a7" order="1"/>
<bond atomRefs2="a2 a3" order="2"/>
<bond atomRefs2="a2 a5" order="1"/>
<bond id="b1" atomRefs2="a2 a1" order="1"/>
</bondArray>
<propertyList>
<property title="program">
<scalar>Gaussian 09, Revision C.01</scalar>
</property>
<property dictRef="me:imaginaryFrequencyScaleFactor" default="true">
<scalar>1</scalar>
</property>
<property title="basis">
<scalar>6-311+G(3df,2pd) (5D, 7F)</scalar>
</property>
<property title="File Format">
<scalar>g03</scalar>
</property>
<property title="Energy" dictRef="me:ZPE">
<scalar units="kJ/mol">-97.2</scalar>
</property>
<property title="SpinMultiplicity" dictRef="me:spinMultiplicity">
<scalar>2.00 </scalar>
</property>
<property title="Hessian" dictRef="me:hessian">
<matrix rows="21" matrixType="squareSymmetricLT" units="Hartree/Bohr2"
>0.09120141 -0.25042474 0.85630591 -0.13575041 0.46197914 0.24631727
0.00089716 -0.01833804 -0.01331631 0.87067355 0.01401462 -0.05587441 -
0.01674221 -0.07021953 0.13062438 -0.01012134 0.01878864 0.05736105
0.36001597 0.10715568 0.40990009 -0.00681183 0.01951895 0.00588197 -
0.66336769 -0.00362286 -0.40054365 0.77354528 -0.00411145 0.01931692
0.01026237 -0.00526017 -0.03885001 -0.03204455 -0.02209921 0.02576688 -
0.01055948 0.01218002 -0.00152693 -0.38992610 -0.02438491 -0.31744883
0.40942551 0.00799600 0.28876161 -0.07813247 0.24950356 0.14143033 -
0.00398917 -0.00969338 0.00694636 0.00487089 0.00369647 0.00413031
0.07746290 0.24575695 -0.83688556 -0.46477222 0.02009527 0.03487561 -
0.02166759 -0.01859626 -0.01375441 -0.01229988 -0.24915727 0.82964305
0.14785897 -0.49442908 -0.30366306 0.01173874 -0.00468865 -0.02027360 -
0.00727851 -0.00252561 -0.00539093 -0.15476910 0.50673389 0.33814653 -
0.00501594 0.00073336 0.00341104 -0.14080939 0.07856353 0.08366301 -
0.10580837 0.02201604 -0.01848367 -0.00051110 0.00203451 0.00248476
0.43660548 -0.00598875 0.02047726 0.01095474 0.06907518 -0.07130355 -
0.09418297 0.01762918 0.00355971 0.02006167 0.00575651 -0.01639377 -
0.00586768 -0.07271858 0.11493039 0.00041996 0.00121481 0.00240659
0.03746189 -0.07312218 -0.12993060 -0.00976062 0.02030463 0.03035818
0.00400851 -0.00759965 -0.00877290 0.08391261 0.09983405 0.26703924 -

0.00279500 -0.00170952 -0.00316045 -0.05960893 -0.01200890 -0.03098430 -
0.00128808 0.00638721 0.00305081 0.00074516 0.00074800 0.00074719 -
0.16474221 -0.04335328 -0.09356691 0.25960842 0.00042118 -0.00161240 -
0.00040320 0.00003775 0.00293155 0.02452245 0.01087747 0.00343706 -
0.00319241 -0.00020457 0.00136610 0.00019140 -0.01371121 -0.06849790 -
0.02596128 -0.01772261 0.49574435 0.00940574 -0.00035891 -0.00101304 -
0.00643963 0.00996250 0.00030763 0.00255170 -0.00288727 0.00524769 -
0.00257857 0.00029687 0.00002336 -0.14061425 -0.05030012 -0.14055732
0.17352636 -0.16455731 0.23932028 0.00065667 0.00071643 0.00150383 -
0.00379553 0.00296653 -0.00897606 -0.00114020 -0.00062889 0.00236262 -
0.00044621 -0.00088118 -0.00078205 -0.01971848 0.02959974 -0.02247544 -
0.03191938 0.02030198 -0.03585135 0.05636314 0.00033220 -0.00172771 -
0.00127862 0.00460953 -0.00240357 -0.00257166 -0.00370727 0.00052385 -
0.00036048 0.00009868 0.00114898 0.00058573 -0.01691765 0.01722786 -
0.01467037 0.06765910 -0.43336876 0.20784425 -0.05207460 0.41859936 -
0.00125344 0.00062540 0.00011812 0.00046545 0.00181978 0.00008427 -
0.00027640 -0.00110557 -0.00000080 0.00083215 -0.00069141 -0.00006940 -
0.01437350 0.01950032 -0.02054319 -0.04961270 0.16940034 -0.10332859
0.06421844 -0.18954885 0.12373959 </matrix>
</property>
<property title="Vibrational Frequencies" dictRef="me:vibFreqs">
<array units="cm-1">63.03 156.90 207.07 250.07 280.53 383.65 443.16 634.20
1045.37 1093.91 1455.29 1948.80 2147.71 3820.93 </array>
</property>
<property title="ImaginaryFrequency" dictRef="me:imFreqs">
<scalar units="cm-1">302.54 </scalar>
</property>
<property dictRef="me:epsilon">
<scalar>216.11</scalar>
</property>
<property dictRef="me:sigma">
<scalar>4.6</scalar>
</property>
<property title="Rotational Constants" dictRef="me:rotConsts">
<array units="cm-1">0.160 0.104 0.065 </array>
</property>
<property title="Symmetry Number" dictRef="me:symmetryNumber">
<scalar>1 </scalar>
</property>
</propertyList>
<me:ExtraDOSCMethod xsi:type="me:HinderedRotorQM1D">
<me:bondRef>b1</me:bondRef>
<me:HinderedRotorPotential format="numerical" units="kJ/mol"
expansionSize="7">
<me:PotentialPoint angle=" 0 " potential=" 0 "/>
<me:PotentialPoint angle=" 36 " potential="
2.553012571 "/>

<me:PotentialPoint angle=" 72 " potential=" 9.836847953 "/>
<me:PotentialPoint angle=" 108 " potential=" 15.99605041 "/>
<me:PotentialPoint angle=" 144 " potential=" 13.10070677 "/>
<me:PotentialPoint angle=" 180 " potential=" 9.028514264 "/>
<me:PotentialPoint angle=" 216 " potential=" 4.512725153 "/>
<me:PotentialPoint angle=" 252 " potential=" 2.161697548 "/>
<me:PotentialPoint angle=" 288 " potential=" 5.380555297 "/>
<me:PotentialPoint angle=" 324 " potential=" 2.629976477 "/>
<me:PotentialPoint angle=" 360 " potential=" 0.000115522 "/>

</me:HinderedRotorPotential>
</me:ExtraDOSCMethod>
<me:ExtraDOSCMethod xsi:type="me:HinderedRotorQM1D">
<me:bondRef>b2</me:bondRef>
<me:HinderedRotorPotential format="numerical" units="kJ/mol" expansionSize="7">
<me:PotentialPoint angle=" 0 " potential=" 0 "/>
<me:PotentialPoint angle=" 36 " potential=" 3.162758691 "/>
<me:PotentialPoint angle=" 72 " potential=" 9.856187386 "/>
<me:PotentialPoint angle=" 108 " potential=" 13.47909383 "/>
<me:PotentialPoint angle=" 144 " potential=" 9.563252475 "/>
<me:PotentialPoint angle=" 180 " potential=" 2.898108291 "/>
<me:PotentialPoint angle=" 216 " potential=" 0.00663989 "/>
<me:PotentialPoint angle=" 252 " potential=" 2.95864707 "/>
<me:PotentialPoint angle=" 288 " potential=" 6.291454144 "/>
<me:PotentialPoint angle=" 324 " potential=" 4.78536495 "/>
<me:PotentialPoint angle=" 360 " potential=" 0 "/>
</me:HinderedRotorPotential>
</me:ExtraDOSCMethod>

```
</molecule>

<molecule id="O2">
  <propertyList>
    <property title="Energy" dictRef="me:ZPE">
      <scalar units="kJ/mol" >0</scalar>
    </property>
    <property dictRef="me:rotConsts">
      <array units="cm-1">1.44914</array>
    </property>
    <property dictRef="me:symmetryNumber">
      <scalar>2</scalar>
    </property>
    <property dictRef="me:frequenciesScaleFactor">
      <scalar>0.9854</scalar>
    </property>
    <property dictRef="me:vibFreqs">
      <array units="cm-1">1665.4292</array>
    </property>
    <property dictRef="me:MW">
      <scalar units="amu">32</scalar>
    </property>
    <property dictRef="me:spinMultiplicity">
      <scalar>3</scalar>
    </property>
  </propertyList>
</molecule>

<molecule id="OH">
  <propertyList>
    <property title="Energy" dictRef="me:ZPE">
      <scalar units="kJ/mol">-167.36</scalar>
    </property>
  </propertyList>
</molecule>

<molecule id="N2" description="Nitrogen">
  <propertyList>
    <property dictRef="me:epsilon">
      <scalar>82.0</scalar>
    </property>
    <property dictRef="me:sigma">
      <scalar>3.74</scalar>
    </property>
    <property dictRef="me:MW">
      <scalar units="amu">28.0</scalar>
    </property>
```

```
    </propertyList>
  </molecule>

</moleculeList>
<reactionList>
  <reaction id="R1">
    <reactant>
      <molecule ref="OH_i" role="modelled"/>
    </reactant>
    <reactant>
      <molecule ref="Glyoxal" role="excessReactant"/>
    </reactant>
    <product>
      <molecule ref="C1" role="modelled"/>
    </product>
    <me:MCRCMethod xsi:type="me:MesmerILT">
      <me:preExponential units="cm3molecule-1s-1">2.0e-11</me:preExponential>
      <me:activationEnergy units="kJ/mol">0</me:activationEnergy>
      <me:nInfinity>0</me:nInfinity>
    </me:MCRCMethod>
  </reaction>
  <reaction id="R2_2">
    <reactant>
      <molecule ref="C1" role="modelled"/>
    </reactant>
    <product>
      <molecule ref="C2" role="modelled"/>
    </product>
    <transitionState>
      <molecule ref="TS1" role="transitionState"/>
    </transitionState>
    <me:MCRCMethod name="RRKM"/>
  </reaction>
  <reaction id="R3">
    <product>
      <molecule ref="C2" role="modelled"/>
    </product>
    <reactant>
      <molecule ref="HC(O)CO" role="modelled"/>
    </reactant>
    <reactant>
      <molecule ref="H2O" role="excessReactant"/>
    </reactant>
    <me:FragmentDist xsi:type="GaussianFrag">
        <me:muIntercept>-2685.4603641118783</me:muIntercept>
                <me:muGradient>0.50158003</me:muGradient>
        <me:sigmaTwoIntercept>-1122.110663126146</me:sigmaTwoIntercept>
```

<me:sigmaTwoGradient>0.13401113</me:sigmaTwoGradient>
<me:sigmaIntercept>-132.00846000618344</me:sigmaIntercept>
<me:sigmaGradient>0.053485</me:sigmaGradient>
</me:FragmentDist>
<me:excessReactantConc>1E14</me:excessReactantConc>
<me:MCRCMethod xsi:type="me:MesmerILT">
<me:preExponential units="cm3molecule-1s-1">2.0e-10</me:preExponential>
<me:activationEnergy units="kJ/mol">0</me:activationEnergy>
<me:nInfinity >0.0</me:nInfinity>
</me:MCRCMethod>
</reaction>

<reaction id="R4">
<reactant>
<molecule ref="HC(O)CO" role="modelled"/>
</reactant>
<product>
<molecule ref="CO" role="sink"/>
</product>
<product>
<molecule ref="HCO" role="sink"/>
</product>
<transitionState>
<molecule ref="TS2" role="transitionState"/>
</transitionState>
<me:MCRCMethod name="RRKM"/>
</reaction>
<reaction id="R2">
<reactant>
<molecule ref="HC(O)CO" role="modelled"/>
</reactant>
<reactant>
<molecule ref="O2" role="excessReactant"/>
</reactant>
<product>
<molecule ref="R02" role="modelled"/>
</product>
<me:MCRCMethod xsi:type="MesmerILT">
<me:preExponential upper="5-12" lower="1E-14" stepsize="1E-15" referenceTemperature="298." >3.1E-13</me:preExponential>
<me:activationEnergy units="kJ/mol">6</me:activationEnergy>
<me:nInfinity >0</me:nInfinity>
</me:MCRCMethod>
</reaction>
<reaction id="R6">
<reactant>
<molecule ref="R02" role="modelled"/>

```
      </reactant>
      <product>
        <!--<molecule ref="QOOH" role="modelled"/>-->
        <molecule ref="QOOH" role="sink"/>
      </product>
      <transitionState>
        <molecule ref="TS4" role="transitionState"/>
      </transitionState>
      <me:MCRCMethod name="RRKM"/>
    </reaction>
    <!--<reaction id="R7">
      <reactant>
        <molecule ref="QOOH" role="modelled"/>
      </reactant>
      <product>
        <molecule ref="OH" role="sink"/>
      </product>
      <TransitionState>
        <molecule ref="TS5" role="transitionState"/>
      </TransitionState>
      <me:MCRCMethod>RRKM</me:MCRCMethod>
    </reaction>-->

  </reactionList>
  <me:conditions>
    <me:bathGas>N2</me:bathGas>
    <me:PTs>


         <me:PTpair units="Torr" P="80" T="295" refReactionExcess="R2"
excessReactantConc="1.547E+17" precision="dd" >
        <me:experimentalYield ref="QOOH" error="0.032" yieldTime="1.e-
02">0.0508</me:experimentalYield>
      </me:PTpair>
      <me:PTpair units="Torr" P="80" T="295" refReactionExcess="R2"
excessReactantConc="3.265E+17" precision="dd" >
        <me:experimentalYield ref="QOOH" error="0.034" yieldTime="1.e-
02">0.135</me:experimentalYield>
      </me:PTpair>
      <me:PTpair units="Torr" P="80" T="295" refReactionExcess="R2"
excessReactantConc="5.866E+17" precision="dd" >
        <me:experimentalYield ref="QOOH" error="0.036" yieldTime="1.e-
02">0.18893</me:experimentalYield>
      </me:PTpair>
      <me:PTpair units="Torr" P="80" T="295" refReactionExcess="R2"
excessReactantConc="9.24E+17" precision="dd" >
```

<me:experimentalYield ref="QOOH" error="0.036" yieldTime="1.e-02">0.21629</me:experimentalYield>
</me:PTpair>
<me:PTpair units="Torr" P="80" T="295" refReactionExcess="R2" excessReactantConc="13.2E+17" precision="dd" >
<me:experimentalYield ref="QOOH" error="0.041" yieldTime="1.e-02">0.2378</me:experimentalYield>
</me:PTpair>
<me:PTpair units="Torr" P="40" T="295" refReactionExcess="R2" excessReactantConc="0.2636E+17" precision="dd" >
<me:experimentalYield ref="QOOH" error="0.031" yieldTime="1.e-02">0.03818</me:experimentalYield>
</me:PTpair>
<me:PTpair units="Torr" P="40" T="295" refReactionExcess="R2" excessReactantConc="1.129E+17" precision="dd" >
<me:experimentalYield ref="QOOH" error="0.032" yieldTime="1.e-02">0.06349</me:experimentalYield>
</me:PTpair>
<me:PTpair units="Torr" P="40" T="295" refReactionExcess="R2" excessReactantConc="2.13259E+17" precision="dd" >
<me:experimentalYield ref="QOOH" error="0.034" yieldTime="1.e-02">0.155</me:experimentalYield>
</me:PTpair>
<me:PTpair units="Torr" P="40" T="295" refReactionExcess="R2" excessReactantConc="2.174E+17" precision="dd" >
<me:experimentalYield ref="QOOH" error="0.037" yieldTime="1.e-02">0.17117</me:experimentalYield>
</me:PTpair>
<me:PTpair units="Torr" P="40" T="295" refReactionExcess="R2" excessReactantConc="4.056E+17" precision="dd" >
<me:experimentalYield ref="QOOH" error="0.038" yieldTime="1.e-02">0.20193</me:experimentalYield>
</me:PTpair>
<me:PTpair units="Torr" P="40" T="295" refReactionExcess="R2" excessReactantConc="4.93E+17" precision="dd" >
<me:experimentalYield ref="QOOH" error="0.04" yieldTime="1.e-02">0.22995</me:experimentalYield>
</me:PTpair>
<me:PTpair units="Torr" P="40" T="295" refReactionExcess="R2" excessReactantConc="6.93E+17" precision="dd" >
<me:experimentalYield ref="QOOH" error="0.039" yieldTime="1.e-02">0.2238</me:experimentalYield>
</me:PTpair>
<me:PTpair units="Torr" P="40" T="295" refReactionExcess="R2" excessReactantConc="12.88E+17" precision="dd" >
<me:experimentalYield ref="QOOH" error="0.04" yieldTime="1.e-02">0.26005</me:experimentalYield>

</me:PTpair>
<me:PTpair units="Torr" P="20" T="295" refReactionExcess="R2" excessReactantConc="0.56298E+17" precision="dd" >
<me:experimentalYield ref="QOOH" error="0.033" yieldTime="1.e-02">0.105</me:experimentalYield>
</me:PTpair>
<me:PTpair units="Torr" P="20" T="295" refReactionExcess="R2" excessReactantConc="0.939E+17" precision="dd" >
<me:experimentalYield ref="QOOH" error="0.039" yieldTime="1.e-02">0.166</me:experimentalYield>
</me:PTpair>
<me:PTpair units="Torr" P="20" T="295" refReactionExcess="R2" excessReactantConc="2.509E+17" precision="dd" >
<me:experimentalYield ref="QOOH" error="0.037" yieldTime="1.e-02">0.215</me:experimentalYield>
</me:PTpair>
<me:PTpair units="Torr" P="20" T="295" refReactionExcess="R2" excessReactantConc="3.72E+17" precision="dd" >
<me:experimentalYield ref="QOOH" error="0.037" yieldTime="1.e-02">0.188</me:experimentalYield>
</me:PTpair>
<me:PTpair units="Torr" P="20" T="295" refReactionExcess="R2" excessReactantConc="4.12E+17" precision="dd" >
<me:experimentalYield ref="QOOH" error="0.04" yieldTime="1.e-02">0.23972</me:experimentalYield>
</me:PTpair>
<me:PTpair units="Torr" P="20" T="295" refReactionExcess="R2" excessReactantConc="6.39E+17" precision="dd" >
<me:experimentalYield ref="QOOH" error="0.042" yieldTime="1.e-02">0.25785</me:experimentalYield>
</me:PTpair>
<me:PTpair units="Torr" P="10" T="295" refReactionExcess="R2" excessReactantConc="0.4067E+17" precision="dd" >
<me:experimentalYield ref="QOOH" error="0.033" yieldTime="1.e-02">0.06035</me:experimentalYield>
</me:PTpair>
<me:PTpair units="Torr" P="10" T="295" refReactionExcess="R2" excessReactantConc="0.57057E+17" precision="dd" >
<me:experimentalYield ref="QOOH" error="0.034" yieldTime="1.e-02">0.11838</me:experimentalYield>
</me:PTpair>
<me:PTpair units="Torr" P="10" T="295" refReactionExcess="R2" excessReactantConc="1.4129E+17" precision="dd" >
<me:experimentalYield ref="QOOH" error="0.036" yieldTime="1.e-02">0.18617</me:experimentalYield>
</me:PTpair>

<me:PTpair units="Torr" P="10" T="295" refReactionExcess="R2" excessReactantConc="2.4672E+17" precision="dd" >
<me:experimentalYield ref="QOOH" error="0.036" yieldTime="1.e-02">0.1974</me:experimentalYield>
</me:PTpair>
<me:PTpair units="Torr" P="10" T="295" refReactionExcess="R2" excessReactantConc="3.029E+17" precision="dd" >
<me:experimentalYield ref="QOOH" error="0.044" yieldTime="1.e-02">0.24264</me:experimentalYield>
</me:PTpair>
<me:PTpair units="Torr" P="5" T="295" refReactionExcess="R2" excessReactantConc="1.507E+17" precision="dd" >
<me:experimentalYield ref="QOOH" error="0.038" yieldTime="1.e-02">0.214</me:experimentalYield>
</me:PTpair>
<me:PTpair units="Torr" P="5" T="295" refReactionExcess="R2" excessReactantConc="1.49E+17" precision="dd" >
<me:experimentalYield ref="QOOH" error="0.045" yieldTime="1.e-02">0.218</me:experimentalYield>
</me:PTpair>
<me:PTpair units="Torr" P="5" T="295" refReactionExcess="R2" excessReactantConc="1E+17" precision="dd" >
<me:experimentalYield ref="QOOH" error="0.037" yieldTime="1.e-02">0.176</me:experimentalYield>
</me:PTpair>
<me:PTpair units="Torr" P="5" T="295" refReactionExcess="R2" excessReactantConc="0.58E+17" precision="dd" >
<me:experimentalYield ref="QOOH" error="0.036" yieldTime="1.e-02">0.12917</me:experimentalYield>
</me:PTpair>
<me:PTpair units="Torr" P="5" T="295" refReactionExcess="R2" excessReactantConc="0.5714E+17" precision="dd" >
<me:experimentalYield ref="QOOH" error="0.037" yieldTime="1.e-02">0.13369</me:experimentalYield>
</me:PTpair>
<me:PTpair units="Torr" P="5" T="295" refReactionExcess="R2" excessReactantConc="0.191E+17" precision="dd" >
<me:experimentalYield ref="QOOH" error="0.032" yieldTime="1.e-02">0.06</me:experimentalYield>
</me:PTpair>

<me:PTpair units="Torr" P="80" T="212" refReactionExcess="R2" excessReactantConc="0.0183E+17" precision="qd" >
<me:experimentalYield ref="QOOH" error="0.03391" yieldTime="1.e-02">0.1</me:experimentalYield>
</me:PTpair>

<me:PTpair units="Torr" P="80" T="212" refReactionExcess="R2" excessReactantConc="0.0517E+17" precision="qd" >
<me:experimentalYield ref="QOOH" error="0.03527" yieldTime="1.e-02">0.125</me:experimentalYield>
</me:PTpair>
<me:PTpair units="Torr" P="80" T="212" refReactionExcess="R2" excessReactantConc="0.13E+17" precision="qd" >
<me:experimentalYield ref="QOOH" error="0.03875" yieldTime="1.e-02">0.216</me:experimentalYield>
</me:PTpair>
<me:PTpair units="Torr" P="80" T="212" refReactionExcess="R2" excessReactantConc="0.304E+17" precision="qd" >
<me:experimentalYield ref="QOOH" error="0.0412" yieldTime="1.e-02">0.296</me:experimentalYield>
</me:PTpair>
<me:PTpair units="Torr" P="80" T="212" refReactionExcess="R2" excessReactantConc="0.613E+17" precision="qd" >
<me:experimentalYield ref="QOOH" error="0.0443" yieldTime="1.e-02">0.352</me:experimentalYield>
</me:PTpair>
<me:PTpair units="Torr" P="80" T="212" refReactionExcess="R2" excessReactantConc="1.08E+17" precision="qd" >
<me:experimentalYield ref="QOOH" error="0.0468" yieldTime="1.e-02">0.3339</me:experimentalYield>
</me:PTpair>
<me:PTpair units="Torr" P="80" T="212" refReactionExcess="R2" excessReactantConc="1.28E+17" precision="qd" >
<me:experimentalYield ref="QOOH" error="0.0482" yieldTime="1.e-02">0.36635</me:experimentalYield>
</me:PTpair>
<me:PTpair units="Torr" P="40" T="212" refReactionExcess="R2" excessReactantConc="0.0569E+17" precision="qd" >
<me:experimentalYield ref="QOOH" error="0.03681" yieldTime="1.e-02">0.159</me:experimentalYield>
</me:PTpair>
<me:PTpair units="Torr" P="40" T="212" refReactionExcess="R2" excessReactantConc="0.0575E+17" precision="qd" >
<me:experimentalYield ref="QOOH" error="0.03609" yieldTime="1.e-02">0.223</me:experimentalYield>
</me:PTpair>
<me:PTpair units="Torr" P="40" T="212" refReactionExcess="R2" excessReactantConc="0.13E+17" precision="qd" >
<me:experimentalYield ref="QOOH" error="0.0416" yieldTime="1.e-02">0.327</me:experimentalYield>
</me:PTpair>
<me:PTpair units="Torr" P="40" T="212" refReactionExcess="R2" excessReactantConc="0.13E+17" precision="qd" >

<me:experimentalYield ref="QOOH" error="0.03844" yieldTime="1.e-02">0.314</me:experimentalYield>
</me:PTpair>
<me:PTpair units="Torr" P="40" T="212" refReactionExcess="R2" excessReactantConc="0.396E+17" precision="qd" >
<me:experimentalYield ref="QOOH" error="0.0455" yieldTime="1.e-02">0.38</me:experimentalYield>
</me:PTpair>
<me:PTpair units="Torr" P="40" T="212" refReactionExcess="R2" excessReactantConc="0.762E+17" precision="qd" >
<me:experimentalYield ref="QOOH" error="0.0529" yieldTime="1.e-02">0.367</me:experimentalYield>
</me:PTpair>
<me:PTpair units="Torr" P="40" T="212" refReactionExcess="R2" excessReactantConc="1.42E+17" precision="qd" >
<me:experimentalYield ref="QOOH" error="0.0497" yieldTime="1.e-02">0.392</me:experimentalYield>
</me:PTpair>
<me:PTpair units="Torr" P="20" T="212" refReactionExcess="R2" excessReactantConc="0.0692E+17" precision="qd" >
<me:experimentalYield ref="QOOH" error="0.03665" yieldTime="1.e-02">0.188</me:experimentalYield>
</me:PTpair>
<me:PTpair units="Torr" P="20" T="212" refReactionExcess="R2" excessReactantConc="0.248E+17" precision="qd" >
<me:experimentalYield ref="QOOH" error="0.042" yieldTime="1.e-02">0.318</me:experimentalYield>
</me:PTpair>
<me:PTpair units="Torr" P="20" T="212" refReactionExcess="R2" excessReactantConc="0.502E+17" precision="qd" >
<me:experimentalYield ref="QOOH" error="0.0421" yieldTime="1.e-02">0.319</me:experimentalYield>
</me:PTpair>
<me:PTpair units="Torr" P="20" T="212" refReactionExcess="R2" excessReactantConc="0.545E+17" precision="qd" >
<me:experimentalYield ref="QOOH" error="0.0433" yieldTime="1.e-02">0.355</me:experimentalYield>
</me:PTpair>
<me:PTpair units="Torr" P="20" T="212" refReactionExcess="R2" excessReactantConc="0.545E+17" precision="qd" >
<me:experimentalYield ref="QOOH" error="0.03937" yieldTime="1.e-02">0.389</me:experimentalYield>
</me:PTpair>
<me:PTpair units="Torr" P="20" T="212" refReactionExcess="R2" excessReactantConc="0.869E+17" precision="qd" >
<me:experimentalYield ref="QOOH" error="0.0584" yieldTime="1.e-02">0.379</me:experimentalYield>

</me:PTpair>
<me:PTpair units="Torr" P="20" T="212" refReactionExcess="R2" excessReactantConc="0.869E+17" precision="qd" >
<me:experimentalYield ref="QOOH" error="0.0548" yieldTime="1.e-02">0.411</me:experimentalYield>
</me:PTpair>
<me:PTpair units="Torr" P="20" T="212" refReactionExcess="R2" excessReactantConc="1.23E+17" precision="qd" >
<me:experimentalYield ref="QOOH" error="0.0432" yieldTime="1.e-02">0.361</me:experimentalYield>
</me:PTpair>
<me:PTpair units="Torr" P="10" T="212" refReactionExcess="R2" excessReactantConc="0.00767E+17" precision="qd" >
<me:experimentalYield ref="QOOH" error="0.03252" yieldTime="1.e-02">0.0825</me:experimentalYield>
</me:PTpair>
<me:PTpair units="Torr" P="10" T="212" refReactionExcess="R2" excessReactantConc="0.0502E+17" precision="qd" >
<me:experimentalYield ref="QOOH" error="0.0375" yieldTime="1.e-02">0.158</me:experimentalYield>
</me:PTpair>
<me:PTpair units="Torr" P="10" T="212" refReactionExcess="R2" excessReactantConc="0.0802E+17" precision="qd" >
<me:experimentalYield ref="QOOH" error="0.03616" yieldTime="1.e-02">0.199</me:experimentalYield>
</me:PTpair>
<me:PTpair units="Torr" P="10" T="212" refReactionExcess="R2" excessReactantConc="0.105E+17" precision="qd" >
<me:experimentalYield ref="QOOH" error="0.03782" yieldTime="1.e-02">0.212</me:experimentalYield>
</me:PTpair>
<me:PTpair units="Torr" P="10" T="212" refReactionExcess="R2" excessReactantConc="0.247E+17" precision="qd" >
<me:experimentalYield ref="QOOH" error="0.041" yieldTime="1.e-02">0.296</me:experimentalYield>
</me:PTpair>
<me:PTpair units="Torr" P="10" T="212" refReactionExcess="R2" excessReactantConc="0.745E+17" precision="qd" >
<me:experimentalYield ref="QOOH" error="0.0425" yieldTime="1.e-02">0.345</me:experimentalYield>
</me:PTpair>
<me:PTpair units="Torr" P="10" T="212" refReactionExcess="R2" excessReactantConc="1.07E+17" precision="qd" >
<me:experimentalYield ref="QOOH" error="0.0396" yieldTime="1.e-02">0.33</me:experimentalYield>
</me:PTpair>

<me:PTpair units="Torr" P="5" T="212" refReactionExcess="R2" excessReactantConc="0.0113E+17" precision="qd" >
<me:experimentalYield ref="QOOH" error="0.034" yieldTime="1.e-02">0.116</me:experimentalYield>
</me:PTpair>
<me:PTpair units="Torr" P="5" T="212" refReactionExcess="R2" excessReactantConc="0.0356E+17" precision="qd" >
<me:experimentalYield ref="QOOH" error="0.03812" yieldTime="1.e-02">0.181</me:experimentalYield>
</me:PTpair>
<me:PTpair units="Torr" P="5" T="212" refReactionExcess="R2" excessReactantConc="0.156E+17" precision="qd" >
<me:experimentalYield ref="QOOH" error="0.0404" yieldTime="1.e-02">0.282</me:experimentalYield>
</me:PTpair>
<me:PTpair units="Torr" P="5" T="212" refReactionExcess="R2" excessReactantConc="0.368E+17" precision="qd" >
<me:experimentalYield ref="QOOH" error="0.043" yieldTime="1.e-02">0.367</me:experimentalYield>
</me:PTpair>
<me:PTpair units="Torr" P="5" T="212" refReactionExcess="R2" excessReactantConc="1.01E+17" precision="qd" >
<me:experimentalYield ref="QOOH" error="0.0482" yieldTime="1.e-02">0.377</me:experimentalYield>
</me:PTpair>
<me:PTpair units="Torr" P="80" T="250" refReactionExcess="R2" excessReactantConc="0.13905E+17" precision="dd" >
<me:experimentalYield ref="QOOH" error="0.03208" yieldTime="1.e-02">0.0786</me:experimentalYield>
</me:PTpair>
<me:PTpair units="Torr" P="80" T="250" refReactionExcess="R2" excessReactantConc="0.294E+17" precision="dd" >
<me:experimentalYield ref="QOOH" error="0.0372" yieldTime="1.e-02">0.166</me:experimentalYield>
</me:PTpair>
<me:PTpair units="Torr" P="80" T="250" refReactionExcess="R2" excessReactantConc="0.44823E+17" precision="dd" >
<me:experimentalYield ref="QOOH" error="0.03945" yieldTime="1.e-02">0.185</me:experimentalYield>
</me:PTpair>
<me:PTpair units="Torr" P="80" T="250" refReactionExcess="R2" excessReactantConc="0.846E+17" precision="dd" >
<me:experimentalYield ref="QOOH" error="0.0436" yieldTime="1.e-02">0.236</me:experimentalYield>
</me:PTpair>
<me:PTpair units="Torr" P="80" T="250" refReactionExcess="R2" excessReactantConc="1.71E+17" precision="dd" >

<me:experimentalYield ref="QOOH" error="0.0465" yieldTime="1.e-02">0.249</me:experimentalYield>
</me:PTpair>
<me:PTpair units="Torr" P="80" T="250" refReactionExcess="R2" excessReactantConc="2.8136E+17" precision="dd" >
<me:experimentalYield ref="QOOH" error="0.0455" yieldTime="1.e-02">0.294</me:experimentalYield>
</me:PTpair>
<me:PTpair units="Torr" P="80" T="250" refReactionExcess="R2" excessReactantConc="3.21E+17" precision="dd" >
<me:experimentalYield ref="QOOH" error="0.0464" yieldTime="1.e-02">0.272</me:experimentalYield>
</me:PTpair>
<me:PTpair units="Torr" P="40" T="250" refReactionExcess="R2" excessReactantConc="0.2E+17" precision="dd" >
<me:experimentalYield ref="QOOH" error="0.03373" yieldTime="1.e-02">0.07</me:experimentalYield>
</me:PTpair>
<me:PTpair units="Torr" P="40" T="250" refReactionExcess="R2" excessReactantConc="0.44E+17" precision="dd" >
<me:experimentalYield ref="QOOH" error="0.03524" yieldTime="1.e-02">0.16</me:experimentalYield>
</me:PTpair>
<me:PTpair units="Torr" P="40" T="250" refReactionExcess="R2" excessReactantConc="0.81E+17" precision="dd" >
<me:experimentalYield ref="QOOH" error="0.047" yieldTime="1.e-02">0.23</me:experimentalYield>
</me:PTpair>
<me:PTpair units="Torr" P="40" T="250" refReactionExcess="R2" excessReactantConc="1.95E+17" precision="dd" >
<me:experimentalYield ref="QOOH" error="0.04" yieldTime="1.e-02">0.24</me:experimentalYield>
</me:PTpair>
<me:PTpair units="Torr" P="40" T="250" refReactionExcess="R2" excessReactantConc="2.77E+17" precision="dd" >
<me:experimentalYield ref="QOOH" error="0.044" yieldTime="1.e-02">0.27</me:experimentalYield>
</me:PTpair>
<me:PTpair units="Torr" P="20" T="250" refReactionExcess="R2" excessReactantConc="0.201E+17" precision="dd" >
<me:experimentalYield ref="QOOH" error="0.03537" yieldTime="1.e-02">0.11</me:experimentalYield>
</me:PTpair>
<me:PTpair units="Torr" P="20" T="250" refReactionExcess="R2" excessReactantConc="0.414E+17" precision="dd" >
<me:experimentalYield ref="QOOH" error="0.03337" yieldTime="1.e-02">0.148</me:experimentalYield>

</me:PTpair>
<me:PTpair units="Torr" P="20" T="250" refReactionExcess="R2" excessReactantConc="1.07E+17" precision="dd" >
<me:experimentalYield ref="QOOH" error="0.0407" yieldTime="1.e-02">0.241</me:experimentalYield>
</me:PTpair>
<me:PTpair units="Torr" P="20" T="250" refReactionExcess="R2" excessReactantConc="1.64E+17" precision="dd" >
<me:experimentalYield ref="QOOH" error="0.0414" yieldTime="1.e-02">0.275</me:experimentalYield>
</me:PTpair>
<me:PTpair units="Torr" P="20" T="250" refReactionExcess="R2" excessReactantConc="2.49E+17" precision="dd" >
<me:experimentalYield ref="QOOH" error="0.03871" yieldTime="1.e-02">0.265</me:experimentalYield>
</me:PTpair>
<me:PTpair units="Torr" P="20" T="250" refReactionExcess="R2" excessReactantConc="3.18E+17" precision="dd" >
<me:experimentalYield ref="QOOH" error="0.046" yieldTime="1.e-02">0.294</me:experimentalYield>
</me:PTpair>
<me:PTpair units="Torr" P="10" T="250" refReactionExcess="R2" excessReactantConc="0.13E+17" precision="dd" >
<me:experimentalYield ref="QOOH" error="0.03663" yieldTime="1.e-02">0.13</me:experimentalYield>
</me:PTpair>
<me:PTpair units="Torr" P="10" T="250" refReactionExcess="R2" excessReactantConc="0.54E+17" precision="dd" >
<me:experimentalYield ref="QOOH" error="0.0404" yieldTime="1.e-02">0.22</me:experimentalYield>
</me:PTpair>
<me:PTpair units="Torr" P="10" T="250" refReactionExcess="R2" excessReactantConc="1.19E+17" precision="dd" >
<me:experimentalYield ref="QOOH" error="0.043" yieldTime="1.e-02">0.27</me:experimentalYield>
</me:PTpair>
<me:PTpair units="Torr" P="10" T="250" refReactionExcess="R2" excessReactantConc="2.55E+17" precision="dd" >
<me:experimentalYield ref="QOOH" error="0.046" yieldTime="1.e-02">0.28</me:experimentalYield>
</me:PTpair>
<me:PTpair units="Torr" P="5" T="250" refReactionExcess="R2" excessReactantConc="0.08E+17" precision="dd" >
<me:experimentalYield ref="QOOH" error="0.03364" yieldTime="1.e-02">0.0731</me:experimentalYield>
</me:PTpair>

```
    <me:PTpair units="Torr" P="5" T="250" refReactionExcess="R2"
excessReactantConc="0.15857E+17" precision="dd" >
     <me:experimentalYield ref="QOOH" error="0.03585" yieldTime="1.e-
02">0.136</me:experimentalYield>
    </me:PTpair>
    <me:PTpair units="Torr" P="5" T="250" refReactionExcess="R2"
excessReactantConc="0.359E+17" precision="dd" >
     <me:experimentalYield ref="QOOH" error="0.0477" yieldTime="1.e-
02">0.194</me:experimentalYield>
    </me:PTpair>
    <me:PTpair units="Torr" P="5" T="250" refReactionExcess="R2"
excessReactantConc="0.7074E+17" precision="dd" >
     <me:experimentalYield ref="QOOH" error="0.0416" yieldTime="1.e-
02">0.223</me:experimentalYield>
    </me:PTpair>
    <me:PTpair units="Torr" P="5" T="250" refReactionExcess="R2"
excessReactantConc="1.93E+17" precision="dd" >
     <me:experimentalYield ref="QOOH" error="0.0499" yieldTime="1.e-
02">0.281</me:experimentalYield>
    </me:PTpair>
   </me:PTs>
        <me:InitialPopulation>
        <me:molecule population="1.0" ref="OH_i"/>
        </me:InitialPopulation>
  </me:conditions>
  <me:modelParameters>
   <me:grainSize units="cm-1">75</me:grainSize>
   <me:energyAboveTheTopHill>25.0</me:energyAboveTheTopHill>
  </me:modelParameters>

  <me:control>
         <!--><me:calcMethod xsi:type="me:marquardt">
            <me:MarquardtIterations>8</me:MarquardtIterations>
            <me:MarquardtTolerance>0.000001</me:MarquardtTolerance>
            <me:MarquardtDerivDelta>1.e-02</me:MarquardtDerivDelta>
           </me:calcMethod>-->
         <me:printGrainedSpeciesProfile/>
         <me:printSpeciesProfile/>
        <me:printGrainBoltzmann/>
   <me:eigenvalues>1</me:eigenvalues>
  </me:control>

</me:mesmer>
```

S4. References

(1) Shannon, R. J.; Robertson, S. H.; Blitz, M. A.; Seakins, P. W. Bimolecular Reactions of Activated Species: An Analysis of Problematic HC(O)C(O) Chemistry. *Chem. Phys. Lett.* **2016**, *661*, 58–64. https://doi.org/https://doi.org/10.1016/j.cplett.2016.08.055.
